# Loss of Axonal Mitochondria Promotes Tau-Mediated Neurodegeneration and Alzheimer's Disease–Related Tau Phosphorylation Via PAR-1

**DOI:** 10.1371/journal.pgen.1002918

**Published:** 2012-08-30

**Authors:** Kanae Iijima-Ando, Michiko Sekiya, Akiko Maruko-Otake, Yosuke Ohtake, Emiko Suzuki, Bingwei Lu, Koichi M. Iijima

**Affiliations:** 1 Laboratory of Neurogenetics and Pathobiology, Department of Neuroscience, Farber Institute for Neurosciences, Thomas Jefferson University, Philadelphia, Pennsylvania, United States of America; 2 Laboratory of Neurobiology and Genetics, Department of Neuroscience, Farber Institute for Neurosciences, Thomas Jefferson University, Philadelphia, Pennsylvania, United States of America; 3 Gene Network Laboratory, National Institute of Genetics, Mishima, Shizuoka, Japan; 4 Department of Genetics, SOKENDAI, Mishima, Shizuoka, Japan; 5 Department of Pathology, Stanford University School of Medicine, Stanford, California, United States of America; University of Minnesota, United States of America

## Abstract

Abnormal phosphorylation and toxicity of a microtubule-associated protein tau are involved in the pathogenesis of Alzheimer's disease (AD); however, what pathological conditions trigger tau abnormality in AD is not fully understood. A reduction in the number of mitochondria in the axon has been implicated in AD. In this study, we investigated whether and how loss of axonal mitochondria promotes tau phosphorylation and toxicity *in vivo*. Using transgenic *Drosophila* expressing human tau, we found that RNAi–mediated knockdown of milton or Miro, an adaptor protein essential for axonal transport of mitochondria, enhanced human tau-induced neurodegeneration. Tau phosphorylation at an AD–related site Ser262 increased with knockdown of milton or Miro; and partitioning defective-1 (PAR-1), the *Drosophila* homolog of mammalian microtubule affinity-regulating kinase, mediated this increase of tau phosphorylation. Tau phosphorylation at Ser262 has been reported to promote tau detachment from microtubules, and we found that the levels of microtubule-unbound free tau increased by milton knockdown. Blocking tau phosphorylation at Ser262 site by PAR-1 knockdown or by mutating the Ser262 site to unphosphorylatable alanine suppressed the enhancement of tau-induced neurodegeneration caused by milton knockdown. Furthermore, knockdown of milton or Miro increased the levels of active PAR-1. These results suggest that an increase in tau phosphorylation at Ser262 through PAR-1 contributes to tau-mediated neurodegeneration under a pathological condition in which axonal mitochondria is depleted. Intriguingly, we found that knockdown of milton or Miro alone caused late-onset neurodegeneration in the fly brain, and this neurodegeneration could be suppressed by knockdown of *Drosophila* tau or PAR-1. Our results suggest that loss of axonal mitochondria may play an important role in tau phosphorylation and toxicity in the pathogenesis of AD.

## Author Summary

Abnormal phosphorylation and toxicity of a microtubule-associated protein tau are involved in the pathogenesis of Alzheimer's disease (AD). Tau is phosphorylated at multiple sites, and phosphorylation of tau regulates its microtubule binding and physiological functions such as regulation of microtubule stability. Abnormal phosphorylation of tau occurs in the AD brains and is thought to cause tau toxicity; however, what pathological conditions trigger abnormal phosphorylation and toxicity of tau in AD is not fully understood. Since a reduction in the number of mitochondria in the axon has been observed in the AD brains, we investigated whether and how loss of axonal mitochondria promotes tau phosphorylation and toxicity. Using transgenic flies expressing human tau, we found that knockdown of milton or Miro, an adaptor protein essential for axonal transport of mitochondria, enhanced human tau-induced neurodegeneration. This study demonstrates that loss of axonal mitochondria caused by milton knockdown increases tau phosphorylation at an AD–related site through partitioning defective-1 (PAR-1), promotes detachment of tau from microtubules, and enhances tau-mediated neurodegeneration. Our results suggest that loss of axonal mitochondria may play an important role in tau phosphorylation and toxicity in the pathogenesis of AD.

## Introduction

Mitochondria are principal mediators of local ATP supply and Ca^2+^ buffering. In neuronal axons, these requirements need to be addressed locally, and the proper distribution of mitochondria is essential for neuronal functions and survival [1]. Defects in mitochondrial distribution have been observed in the brains of patients suffering from several neurodegenerative diseases including Alzheimer's disease (AD) [2]. Recent studies have shown that the localization of mitochondria to the axon is reduced in neurons in the AD brain, as well as in cellular and animal models of AD [3]–[14]. The reduction in mitochondria in the axon may be due to alterations in mitochondrial fission/fusion [3], [5], [6] and/or due to defects in the axonal transport of mitochondria [4], [6], [8], [10]–[12]. However, how it contributes to the pathogenesis of AD remains elusive.

Tau is a microtubule-associated protein that is expressed in neurons and localizes predominantly in the axons, where it regulates microtubule dynamics. Tau is phosphorylated at a number of sites, and a fine-tuned balance between phosphorylation and dephosphorylation of tau is critical for its physiological functions, such as microtubule stabilization, in the axons [15].

Hyperphosphorylated tau is found in neurofibrillary tangles, the intracellular protein inclusions that are associated with a range of neurodegenerative diseases including AD [15]. In AD brains, tau phosphorylation is abnormally increased at several specific sites, and these changes are associated with tau toxicity [15], [16]. However, the effects of loss of axonal mitochondria on abnormal phosphorylation and toxicity of tau has not been fully elucidated.

Mitochondrial transport is regulated by a series of molecular adaptors that mediate the attachment of mitochondria to molecular motors [17]. In *Drosophila*, mitochondrial transport is facilitated by milton and Miro, which regulate mitochondrial attachment to microtubules via kinesin heavy chain [18], [19]. In mammals, two isoforms of milton (OIP106 and GRIF1) and Miro (Miro1 and Miro2) have been identified and are proposed to act in a similar manner [20]. In *Drosophila*, in the absence of milton or Miro, synaptic terminals and axons lack mitochondria, although mitochondria are numerous in the neuronal cell body [18], [21].

In this study, using *Drosophila* as a model system, we investigated the effects of knockdown of milton or Miro, an adaptor protein essential for axonal transport of mitochondria, on tau phosphorylation and toxicity. We demonstrate that loss of axonal mitochondria caused by milton knockdown increases tau phosphorylation at Ser262 through PAR-1, promotes detachment of tau from microtubules, and enhances tau-mediated neurodegeneration.

## Results

### Knockdown of milton or Miro significantly enhances human tau-mediated neurodegeneration

To test whether loss of axonal mitochondria enhances human tau toxicity *in vivo*, we used transgenic *Drosophila* expressing human tau [22]. Wild-type human 0N4R tau, which has four tubulin-binding domains (R) and no N-terminal insert (N), was expressed in fly eyes using the GAL4/UAS system [23] with the pan-retinal gmr-GAL4 driver. Expression of human tau causes age-dependent and progressive neurodegeneration in the lamina, the first synaptic neuropil of the optic lobe containing photoreceptor axons: degeneration in the lamina is undetectable or very mild in flies at 3-day-old, while it is prominent at 10-day-old (Figure S1A and S1B; S1D, quantification).

It has been reported that overexpression of tau alone can reduce anterograde transport of a variety of kinesin cargos, including mitochondria [4], [9], [11], [12], [24]. We examined whether tau expression alone causes the loss of mitochondria at the synaptic terminals of young tau flies by electron microscopy. Mitochondria were observed in the synaptic terminals of photoreceptor neurons expressing tau at 3-day-old (Figure S2), suggesting that, under our experimental conditions, severe defects in microtubule-dependent transport are not occurred in the young flies expressing human tau.

**Figure 1:**
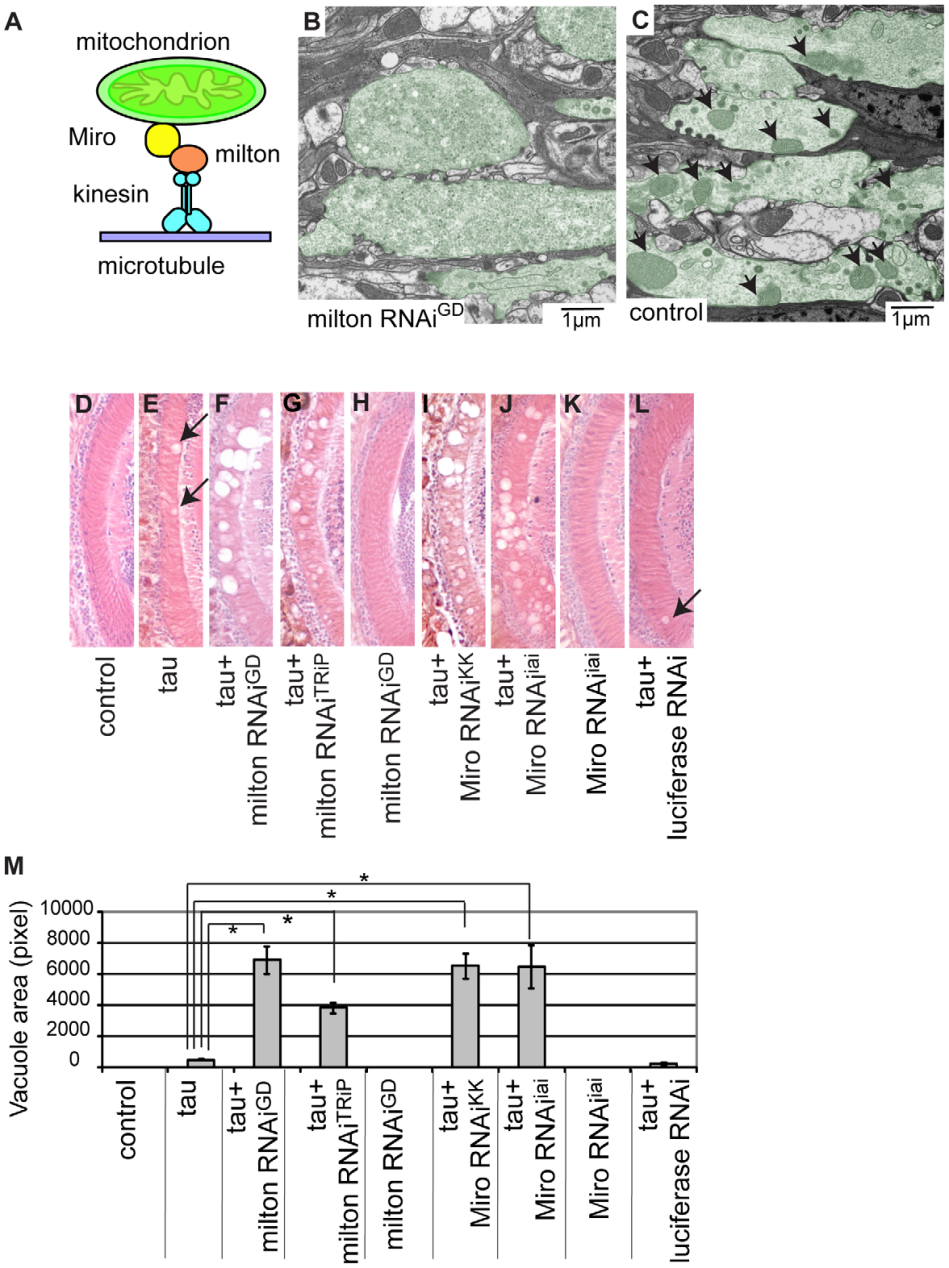
RNAi–mediated knockdown of milton or Miro enhances human tau-induced neurodegeneration. (A) The mitochondrial transport machinery. (B–C) Transmission electron micrographs of presynaptic terminals in the lamina of flies expressing milton RNAi with the gmr-GAL4 driver (B) and control flies bearing the gmr-GAL4 driver only (C). Presynaptic terminals in B and C are colored green to accentuate the structures. Arrows in C indicate mitochondria in synaptic terminals. Note that synaptic terminals in milton knockdown flies (B) lack mitochondria, while the synaptic terminals of control flies (C) contain mitochondria. Flies are 3 days-after-eclosion (day-old). (D–M) The lamina of control flies bearing the driver only (D), flies expressing tau alone (E), co-expressing tau and milton RNAi^GD^ (F), co-expressing tau and milton RNAi^TRiP^ (G), expressing milton RNAi^GD^ alone (H), co-expressing tau and Miro RNAi^KK^ (I), co-expressing tau and Miro RNAi^iai^ (J), expressing Miro RNAi^iai^ alone (K), or co-expressing tau and luciferase RNAi (L). In E and L, neurodegeneration is indicated by arrows. (M) Quantification of neurodegeneration, mean ± SEM, n = 10–33. *, p<0.05, Student's t-test. Flies were 3 days-after-eclosion (day-old).

Milton is a component of an adaptor complex that links mitochondria to kinesin heavy chain and is essential for axonal transport of mitochondria (Figure 1A) [19]. Previously, we have shown that milton RNAi expression effectively reduces milton protein levels, reduces the axonal distribution of mitochondria and increases the mitochondrial localization to the cell body in the fly brain [7]. We confirmed that expression of milton RNAi in fly eyes caused loss of mitochondria in the synaptic terminals of the photoreceptor neurons by electron microscopy analysis. Mitochondria were seldom observed in the synaptic terminals of photoreceptor neurons expressing milton RNAi, while mitochondria were abundant in the synaptic terminals of control flies (compare Figure 1B and 1C). In addition, the presynaptic terminals contained vesicles with a wider range of sizes in the milton knockdown flies than in controls (Figure S3), as previously observed in milton mutant flies [25].

To investigate tau toxicity under the condition in which mitochondria are chronically depleted from the axon, we co-expressed milton RNAi with human tau. We confirmed that milton knockdown caused loss of axonal mitochondria in the neurons expressing tau by electron microscopy (Figure S4). Co-expression of tau with milton RNAi (milton RNAi^GD^) dramatically enhanced neurodegeneration in the lamina at 3-day-old compared to fly eyes expressing tau alone (Figure 1E and 1F; 1M, quantification). In 3-day-old flies, knockdown of milton alone did not cause neurodegeneration (Figure 1H) [21]. To limit the possibility of off-target effects of RNAi, another independent transgenic fly line carrying a milton RNAi that targets a different region of milton (milton RNAi^TRiP^) was used. Expression of this RNAi in neurons reduced milton mRNA levels in the fly brain (Figure S5A) as well as the axonal distribution of mitochondria (Figure S5B). The enhancement of tau-induced neurodegeneration was also observed with milton RNAi^TRiP^ (Figure 1G; 1M, quantification).

We also tested the effect of knockdown of Miro, which is another critical component of the adaptor complex that controls mitochondrial trafficking in the axons [18], [19] (Figure 1A), on tau-mediated neurodegeneration. Expression of Miro RNAi (Miro RNAi^KK^) [26] reduced the axonal distribution of mitochondria in the fly brain (Figure S6) and significantly enhanced tau-induced neurodegeneration (Figure 1I; 1M, quantification). To limit the possibility of off-target effects of RNAi, we generated another independent transgenic fly line carrying Miro RNAi (Miro RNAi^iai^) that targets a different region of Miro. Expression of Miro RNAi^iai^ reduced Miro mRNA levels (Figure S7) and significantly enhanced tau-induced neurodegeneration (Figure 1J; 1M, quantification). Similar to a previous report [21], knockdown of Miro alone did not cause neurodegeneration in 3-day-old flies (Figure 1K).

The enhancement of tau-induced neurodegeneration by milton RNAi or Miro RNAi is not due to non-specific effects of RNAi overexpression, since the expression of an RNAi against firefly luciferase (Figure 1L), as well as the expression of many other RNAis (Figure S8), did not enhance tau-induced neurodegeneration.

Expression of human tau in *Drosophila* eyes reduces the external eye size (Figure S9B), which is due to apoptosis during the larval stage [27]. Genetic screens assessing changes in this phenotype have identified a number of modifiers of tau toxicity [28]–[30]. However, neither milton nor Miro was identified in the previous screens [28]–[30]. We found that knockdown of either milton or Miro did not enhance the tau-induced reduction in external eye size (Figure S9C and S9D). These results indicate that the modifier screen using tau-induced lamina degeneration as a read-out phenotype yields new genes involved in tau-induced neurodegeneration.

Taken together, these results demonstrate that the knockdown of milton or Miro enhances human tau-mediated neurodegeneration.

### Milton knockdown enhances tau-induced axon degeneration without neurofibrillary tangle formation

**Figure 2:**
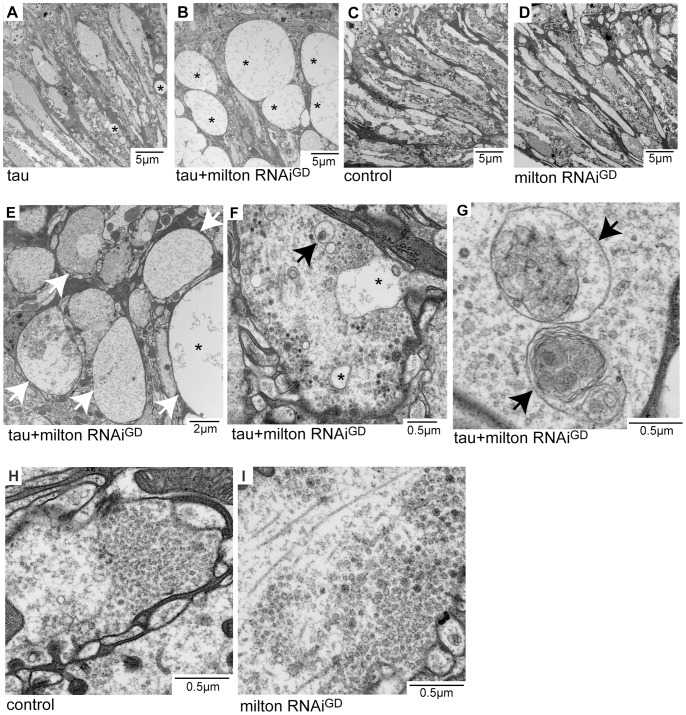
Milton or Miro knockdown enhances tau-induced axon degeneration. Transmission electron micrographs of lamina areas (A–E) and presynaptic terminals of photoreceptor neurons in the lamina (F–I) in flies expressing tau (A), flies co-expressing tau and milton RNAi (B, E, F and G), control flies bearing the gmr-GAL4 driver only (C and H), or flies expressing milton RNAi alone (D and I). Asterisks in A, B, E and F indicate vacuoles. Arrows in E indicate swollen axons. Arrows in F and G indicate autophagic and multivesicular bodies. All flies were 3 days-after-eclosion (day-old).

Neurodegeneration in the lamina in flies expressing tau alone (Figure 2A) or expressing tau and milton RNAi (Figure 2B, 2E, 2F and 2G) at 3-day-old was examined at the ultrastructural level. Axon pathology, including the formation of vacuoles in the axons (asterisks in Figure 2A, 2B and 2E) and swollen axons (arrows in Figure 2E), were observed. In the presynaptic terminals, vacuoles (Figure 2F, asterisks) and the accumulation of autophagic bodies and multivesicular bodies (Figure 2F and 2G, arrows) were observed. These pathological changes were more severe and prominent in the lamina of flies expressing tau and milton RNAi than in flies expressing tau alone. Neurofibrillary tangles were not detected in flies expressing human tau and milton RNAi, indicating that milton knockdown enhances the tau-induced axonopathy without the formation of large tau aggregates. Axonal or presynaptic degeneration was not observed in the control flies (Figure 2C and 2H) or in flies with milton knockdown alone at 3-day-old (Figure 2D and 2I).

### Milton knockdown increases tau phosphorylation levels at AD–related Ser262

**Figure 3:**
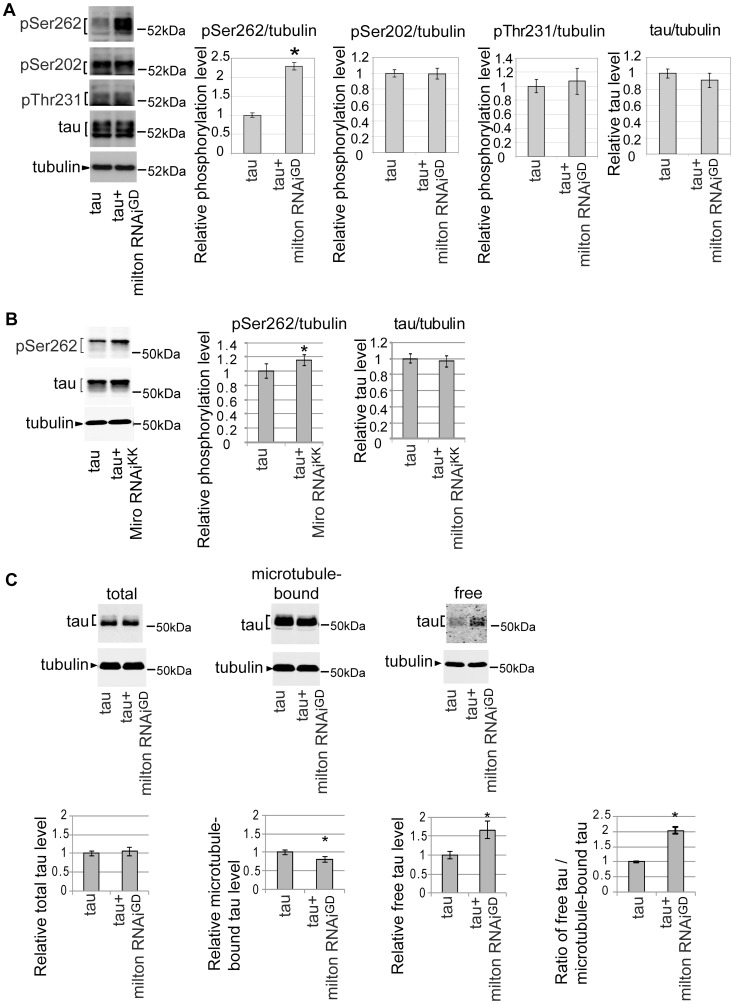
Knockdown of milton or Miro increases tau phosphorylation levels at AD–related Ser262. (A) Milton knockdown increases tau phosphorylation levels at Ser262. Western blots of eyes from flies expressing tau alone (tau) or co-expressing tau and milton RNAi (tau+milton RNAi^GD^). Blots were probed with anti-phospho-Ser262 tau, anti-phospho-Ser202 tau, anti-phospho-Thr231 tau, anti-tau, or anti-tubulin. Tubulin was used as a loading control. Mean ± SD, n = 5; *, p<0.05, Student's t-test. Representative blots are shown. (B) Miro knockdown increases tau phosphorylation levels at Ser262. Western blots of eyes from flies expressing tau alone (tau) or co-expressing tau and Miro RNAi^KK^ (tau+Miro RNAi^KK^). Blots were probed with anti-phospho-Ser262 tau, anti-tau, or anti-tubulin. Tubulin was used as a loading control. Mean ± SD, n = 5; *, p<0.05, Student's t-test. Representative blots are shown. (C) Microtubule binding assay of tau. Proteins were extracted from heads of flies expressing tau alone (tau) or co-expressing tau and milton RNAi (tau+milton RNAi^GD^). Tau or tubulin in the lysate before fractionation (total), the fraction free from microtubules (free) or the fraction containing microtubules (microtubule-bound) were analyzed with western blotting using anti-tau or anti-tubulin. The same amount of protein was loaded to each lane. Each graph displays tau levels relative to control, or the ratio of free tau relative to microtubule-bound tau (Mean ± SD, n = 6; *, p<0.05, Student's t-test). Representative blots are shown. All flies were 3 days-after-eclosion (day-old).

A group of Ser/Thr phosphorylation sites in tau is abnormally phosphorylated in the AD brain [31]. Using well-characterized phospho-tau-specific antibodies, we examined whether milton knockdown affects tau phosphorylation at AD-related sites by Western blotting. The level of tau phosphorylated at Ser262 was significantly increased by milton knockdown (Figure 3A). Knockdown of Miro also increased the levels of tau phosphorylated at Ser262 (Figure 3B). In contrast, tau phosphorylation at the AT8 epitope (phospho-Ser202) or the AT180 epitope (phospho-Thr231) was not significantly altered by milton knockdown (Figure 3A). The levels of total tau were not significantly changed (Figure 3A), indicating that milton knockdown does not cause tau accumulation.

Tau phosphorylation at Ser262 has been reported to reduce tau binding to microtubules [32], [33]. We tested whether milton knockdown alters the binding of tau to microtubules by using microtubule binding assay. Microtubules and microtubule-bound proteins were recovered as the pellet by centrifugation from brains of flies expressing human tau alone or co-expressing human tau and milton RNAi. The pellet (microtubule-bound fraction) and supernatant (microtubule-free fraction) were separated by SDS-PAGE, and tau levels in these fractions were analyzed by Western blotting. Milton knockdown caused a significant reduction in the amount of tau in the pellet and an increase in tau in the supernatant (Figure 3C). This result indicates that milton knockdown reduces tau binding to microtubules and increases the levels of microtubule-unbound, free tau in the fly brain.

### PAR-1 mediates the increase in tau phosphorylation at Ser262 caused by milton knockdown

**Figure 4:**
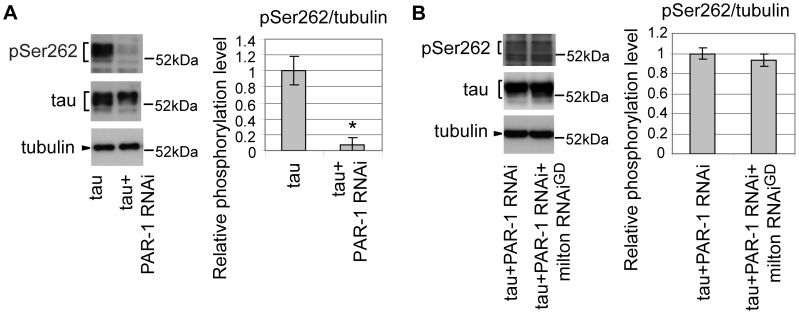
PAR-1 mediates the increase in tau phosphorylation at Ser262 caused by milton knockdown. (A) Western blots of eyes from flies expressing tau alone or co-expressing tau and PAR-1 RNAi. Blots were probed with anti-phospho-Ser262 tau, anti-tau, or anti-tubulin. Mean ± SD, n = 5; *, p<0.05, Student's t-test. (B) Western blots of eyes from flies co-expressing tau and PAR-1 RNAi, or co-expressing tau, PAR-1 RNAi and milton RNAi. Blots were probed with anti-phospho-Ser262, anti-tau, or anti-tubulin. No significant difference was found (mean ± SD, n = 5; p>0.05, Student's t-test). Representative blots are shown. All flies were 3 days-after-eclosion (day-old).

*Drosophila* partitioning defective-1 (PAR-1) and the mammalian homolog of PAR-1, microtubule affinity-regulating kinase (MARK), are reported to phosphorylate tau at Ser262 *in vivo* [34], [35]. RNAi-mediated knockdown of PAR-1 in fly eyes caused a significant reduction in tau phosphorylation at Ser262, suggesting that PAR-1 is the major Ser262 kinase in the fly eye (Figure 4A) [35]. We examined whether blocking PAR-1 activity suppresses the increase in tau phosphorylation at Ser262 caused by milton knockdown. In the PAR-1 knockdown background, milton knockdown did not increase tau phosphorylation levels at Ser262 (Figure 4B), indicating that PAR-1 mediates the increase in tau phosphorylation at Ser262 caused by milton knockdown.

### PAR-1 and tau phosphorylation site Ser262 are critical for the enhancement of tau-induced axon degeneration caused by milton knockdown

**Figure 5:**
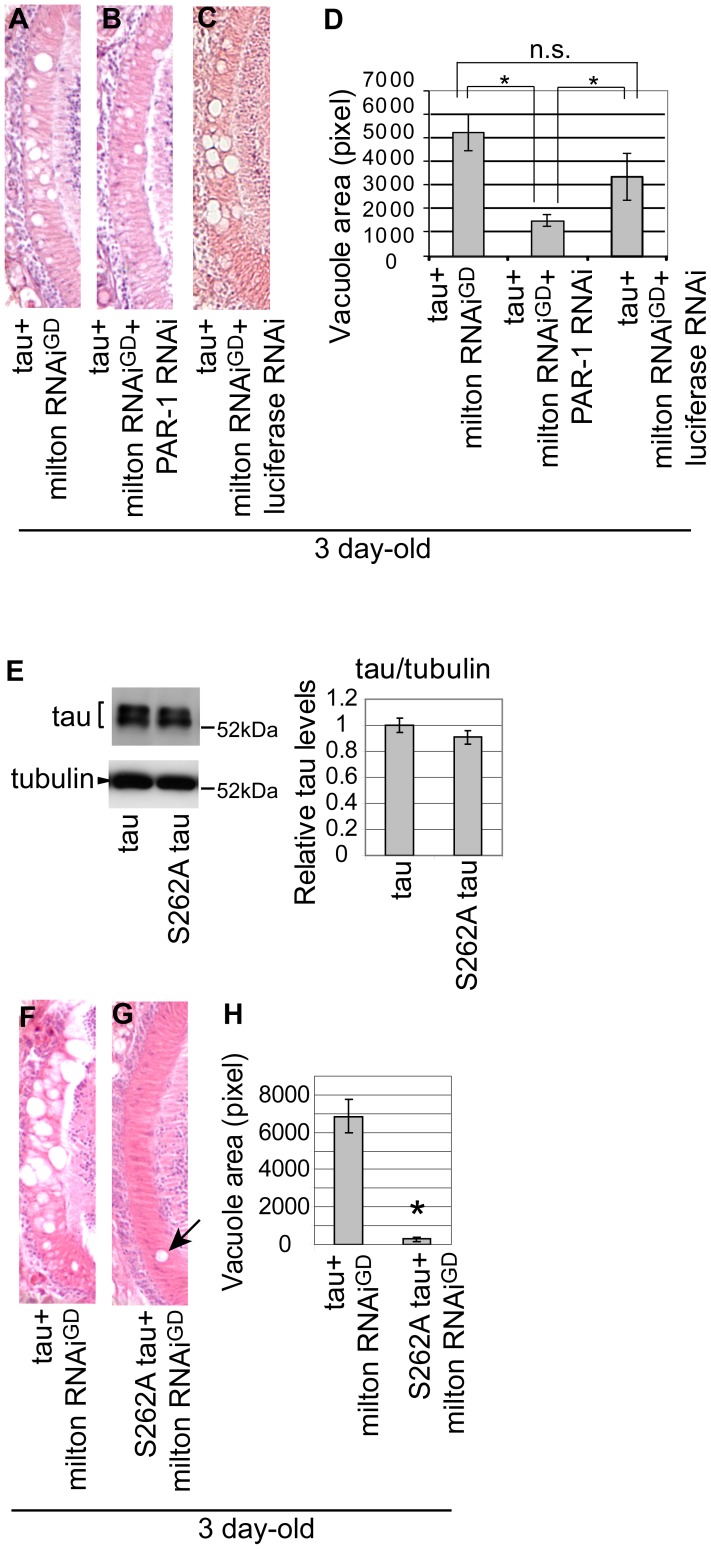
PAR-1 and tau phosphorylation site Ser262 are critical for the enhancement of tau-induced neurodegeneration caused by milton knockdown. (A–C) PAR-1 knockdown suppresses the enhancement of tau-induced neurodegeneration caused by milton knockdown. (A–C) The lamina co-expressing tau and milton RNAi (A), co-expressing tau, milton RNAi and PAR-1 RNAi (B), and co-expressing tau, milton RNAi and luciferase RNAi (C). (D) Quantification of neurodegeneration, mean ± SEM, n = 12. Significant difference was found between tau+milton RNAi^GD^ and tau+ milton RNAi^GD^+PAR-1 RNAi or between tau+milton RNAi^GD^ +luciferase RNAi and tau+ milton RNAi^GD^+PAR-1 RNAi (*, p<0.05, Student's t-test), but not between tau+milton RNAi^GD^ and tau+ milton RNAi^GD^+luciferase RNAi. All flies were 3 days-after-eclosion (day-old). (E–H) The Ser262 phosphorylation site in tau is necessary for the enhancement of tau-induced neurodegeneration caused by milton knockdown. (E) Western blots of the eyes from flies expressing wild-type tau or S262A mutant tau. Blots were probed with anti-tau or anti-tubulin (loading control). Expression levels were similar (mean ± SD, n = 5; p>0.05, Student's t-test). (F–H) The lamina co-expressing wild-type tau and milton RNAi (F), or co-expressing S262A tau and milton RNAi (G). (H) Quantification of neurodegeneration, presented as mean ± SEM, n = 9–12. *, p<0.05, Student's t-test. All flies were 3 days-after-eclosion (day-old).

We investigated the role of tau phosphorylation at Ser262 in the enhancement of tau-induced axon degeneration caused by milton knockdown. We first examined whether PAR-1 knockdown enhances or suppresses tau-induced axon degeneration in the milton knockdown background. RNAi-mediated knockdown of PAR-1 significantly suppressed neurodegeneration in the lamina of flies expressing human tau and milton RNAi (Figure 5A and 5B; 5D, quantification). This effect is not due to titration of the effectiveness of RNAi, since the expression of an RNAi against firefly luciferase did not significantly suppress tau-induced neurodegeneration in the milton knockdown background (Figure 5A and 5C; 5D, quantification).

Next, to determine whether the Ser262 site is required for the knockdown of milton to enhance tau-induced axon degeneration, transgenic flies carrying human tau with the S262A mutation (S262A tau) expressed at the levels similar to the expression of wild-type tau ([36] and Figure 5E) were used. It has been reported that introduction of the S262A mutation dramatically rescues tau-induced reduction in external eye size [35], [36], which is due to apoptosis during the larval stage [27]. Interestingly, we found that expression of S262A tau caused age-dependent neurodegeneration in the lamina similar to that caused by wild type tau: in the flies expressing S262A tau, degeneration in the lamina was undetectable or very mild in flies at 3-day-old, while it was prominent at 10-day-old (Figure S10). Using S262A tau flies, we found that the introduction of the S262A mutation suppressed the enhancement of tau-induced axon degeneration caused by milton knockdown (Figure 5F and 5G; 5H, quantification). Taken together, these results suggest that tau phosphorylation at Ser262 and PAR-1 play a critical role in the enhancement of tau-induced neurodegeneration caused by milton knockdown.

### Knockdown of milton or Miro increases the levels of active PAR-1

Our results demonstrate that knockdown of milton or Miro enhances tau-induced neurodegeneration and increases tau phosphorylation at Ser262. PAR-1 mediates the increase in tau phosphorylation at Ser262, and tau phosphorylation site Ser262 and PAR-1 are critical for the enhancement of tau-induced neurodegeneration caused by milton knockdown. To further investigate the relationship between loss of axonal mitochondria and PAR-1, the effect of knockdown of milton or Miro on PAR-1 activity was examined.

**Figure 6:**
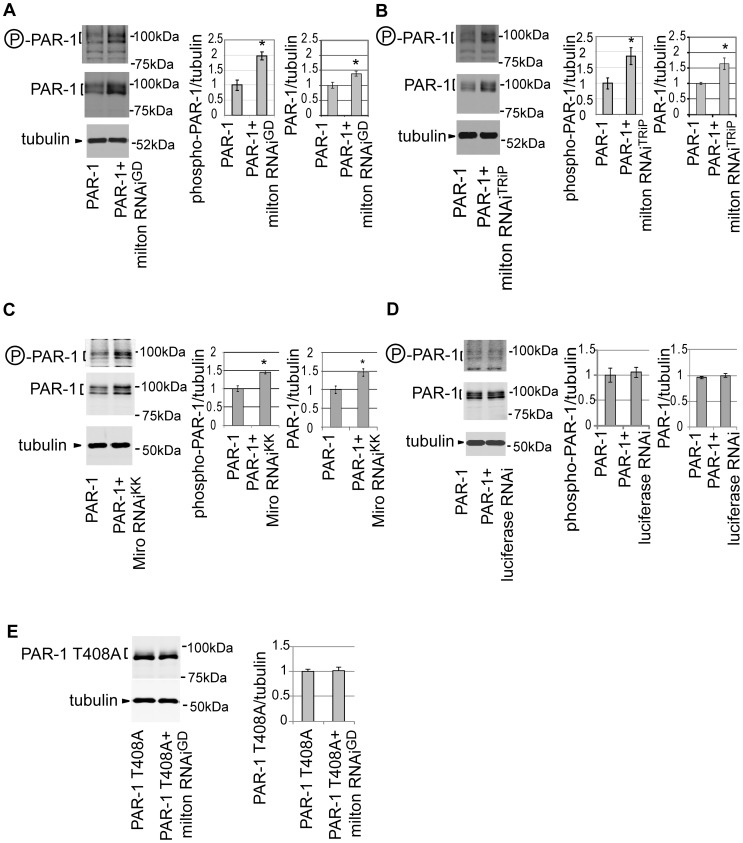
Knockdown of milton or Miro increases the levels of active PAR-1. (A) Western blots of eyes from flies expressing myc-tagged PAR-1 alone or flies co-expressing PAR-1 and milton RNAi^GD^. Blots were probed with anti-phospho-T408 PAR-1 (P-PAR-1), anti-myc (PAR-1), or anti-tubulin. Tubulin was used as loading control. Mean ± SD, n = 5; *, p<0.05, Student's t-test. (B) Western blots of eyes from flies expressing myc-tagged PAR-1 or co-expressing PAR-1 and milton RNAi^TRiP^. Blots were probed with anti-phospho-T408 PAR-1 (P-PAR-1), anti-myc (PAR-1), or anti-tubulin. Mean ± SD, n = 5; *, p<0.05, Student's t-test. (C) Western blots of eyes from flies expressing myc-tagged PAR-1 alone or flies co-expressing PAR-1 and Miro RNAi^KK^. Blots were probed with anti-phospho-T408 PAR-1 (P-PAR-1), anti-myc (PAR-1), or anti-tubulin. Mean ± SD, n = 5; *, p<0.05, Student's t-test. (D) Western blots of eyes from flies expressing myc-tagged PAR-1 alone or flies co-expressing PAR-1 and luciferase RNAi. Blots were probed with anti-phospho-T408 PAR-1 (P-PAR-1), anti-myc (PAR-1), or anti-tubulin. No significant difference was found (mean ± SD, n = 5; p>0.05, Student's t-test). (E) Western blots of eyes from flies expressing myc-tagged PAR-1 T408A alone or flies co-expressing PAR-1 T408A and milton RNAi^GD^. Blots were probed with anti-myc (PAR-1 T408A) or anti-tubulin. No significant difference was found (mean ± SD, n = 5; p>0.05, Student's t-test). Flies were 3 days-after-eclosion (day-old).

To detect active PAR-1, a phospho-specific antibody that recognizes phosphorylated Thr408 of PAR-1, which is important for PAR-1 activity [37], was used. The titer of the antibody is not sufficient to detect endogenous PAR-1 in fly eyes [37], but the antibody recognizes the active form of PAR-1 when PAR-1 is overexpressed [37]. Co-expression of milton RNAi increases the levels of Thr408-phosphorylated PAR-1 in the fly eye (Figure 6A). In addition to the levels of Thr408-phosphorylated PAR-1, we observed an increase in total PAR-1 levels with knockdown of milton (Figure 6A). Similar results were obtained with another milton RNAi line (milton RNAi^TRiP^) (Figure 6B). Furthermore, expression of Miro RNAi also caused an increase in the levels of Thr408-phosphorylated PAR-1 as well as total PAR-1 in the fly eyes (Figure 6C). These effects are not due to non-specific effect of RNAi expression, since the expression of an RNAi against firefly luciferase did not increase either the levels of Thr408-phosphorylated PAR-1 or total PAR-1 (Figure 6D).

A previous report showed that total PAR-1 level increased when it was phosphorylated at Thr408 in *Drosophila* [37]. To examine whether the increase in PAR-1 levels with milton knockdown is Thr408-dependent, transgenic flies carrying PAR-1 with unphosphorylatable alanine mutation at Thr408 (PAR-1 T408A) [37] were used. Milton RNAi coexpression did not increase PAR-1 T408A protein levels (Figure 6E), indicating that Thr408 is important for the increase in PAR-1 levels caused by milton knockdown.

Milton knockdown did not cause non-specific activation of kinases, since it did not increase the level of phosphorylated, active p44mapk in fly eyes (Figure S11). Moreover, milton knockdown did not cause non-specific accumulation of overexpressed proteins, or an increase in the expression of genes under the control of GAL4/UAS system, since it did not increase the levels of total p44mapk, GFP, or amyloid precursor protein expressed via the GAL4/UAS system (Figure S11). Taken together, these results demonstrate that milton knockdown specifically increases the level of active PAR-1.

We also observed that the phenotype induced by PAR-1 overexpression was enhanced by milton knockdown. Overexpression of PAR-1 in fly eyes has been reported to cause eye degeneration [35], and we found that overexpression of PAR-1 caused age-dependent, mild neurodegeneration in the lamina: neurodegeneration in the lamina is undetectable in flies overexpressing PAR-1 at 3-day-old, while it is detectable at 10-day-old (Figure S12A–S12C, S12D, quantification). Co-expression of PAR-1 and milton RNAi caused prominent neurodegeneration in the lamina at 3-day-old, when neither PAR-1 overexpression alone or knockdown of milton alone caused neurodegeneration (Figure S12, S12E–S12G; S12H, quantification). These phenotypic analyses further suggest that milton knockdown increases PAR-1 activity in the eye.

### RNAi–mediated knockdown of milton or Miro alone in neurons causes age-dependent neurodegeneration in the fly brain

**Figure 7:**
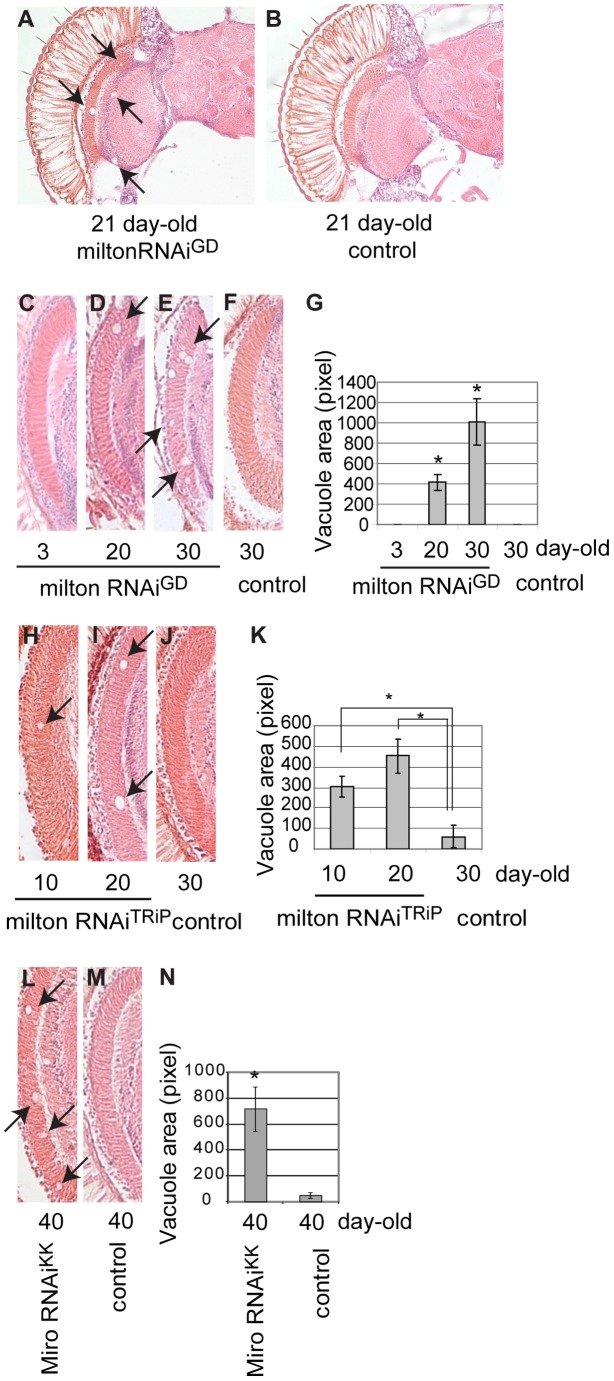
RNAi–mediated knockdown of milton or Miro in neurons causes age-dependent neurodegeneration in the fly brain. (A–B) The brain of flies expressing milton RNAi with a combination of two drivers, the pan-neuronal elav-GAL4 driver and pan-retinal gmr-GAL4 driver (A) and control (luciferase RNAi) (B). Flies are at 21-day-old. Neurodegeneration is indicated by arrows. (C–G) The lamina in flies expressing milton RNAi at 3-day-old (C), 20-day-old (D) or 30-day-old (E), or the lamina of control flies (gmr-GAL4 driver only) at 30-day-old (F). (G) Quantification of neurodegeneration (indicated by arrows in D and E), mean ± SEM, n = 10–12. *, p<0.05, Student's t-test. (H-K) Quantification of neurodegeneration in the lamina in the flies expressing milton RNAi^TRiP^ with a combination of the elav-GAL4 driver and gmr-GAL4 driver at 10-day-old (H) and 20-day-old (I), or neurodegeneration in the lamina of control flies (drivers only) at 30-day-old (J). (K) Quantification of neurodegeneration (arrows in H and I). Mean ± SEM, n = 10–12. *, p<0.05, Student's t-test. (L–N) Quantification of neurodegeneration in the lamina in the flies expressing Miro RNAi^KK^ with the gmr-GAL4 driver (L) or in the lamina of control flies (drivers only) (M) at 40-day-old. (N) Quantification of neurodegeneration (arrows in L). Mean ± SEM, n = 10–12. *, p<0.05, Student's t-test.

Although knockdown of milton or Miro without human tau overexpression did not cause neurodegeneration in the young flies (Figure 1H and 1K), we found that knockdown of milton or Miro alone caused late-onset neurodegeneration in the fly brain. Expression of milton RNAi in the fly eyes and brains with a combination of two GAL4 drivers, the pan-retinal gmr-GAL4 driver and pan-neuronal elav-GAL4 driver, caused age-dependent neurodegeneration (Figure 7A, 21 days-after-eclosion (day-old)). Interestingly, although milton RNAi was expressed in the all neurons in the eye and brain, degeneration was the most prominent in the optic lobe (Figure 7A). No degeneration was observed in the brain in flies expressing an RNAi against firefly luciferase at the same age (Figure 7B, control).

We quantified age-dependent progression of neurodegeneration caused by milton knockdown in the lamina, where neurodegeneration was the most prominent. Degeneration in the lamina is undetectable at 3-day-old, which is in line with the previous observation in milton mutant flies [25]. In contrast, degeneration was observed at 20-day-old and was more prominent at 30-day-old, indicating that milton knockdown causes late-onset and progressive neurodegeneration (Figure 7, compare 7C (3-day-old), 7D (20-day-old) and 7E (30-day-old); G, quantification of vacuole areas). No degeneration was observed in the lamina in control flies at 30-day-old (Figure 7F).

To limit the possibility of off-target effects of RNAi, another independent transgenic fly line carrying a milton RNAi that targets a different region of milton (milton RNAi^TRiP^) was used. Expression of milton RNAi^TRiP^ also caused age-dependent neurodegeneration in the lamina (Figure 7H–7J; 7K, quantification of vacuole areas). Knockdown of Miro in the fly brains also caused neurodegeneration in the lamina in aged flies (Figure 7L–7M; 7N, quantification of vacuole areas). Collectively, these results suggest that loss of axonal mitochondria is sufficient to cause late-onset neurodegeneration.

While our paper is under review, it was reported that knockdown of milton led to progressive axon degeneration in the *Drosophila* wing neurons [38].

### RNAi–mediated knockdown of *Drosophila* tau or PAR-1 suppresses milton knockdown-induced neurodegeneration

**Figure 8:**
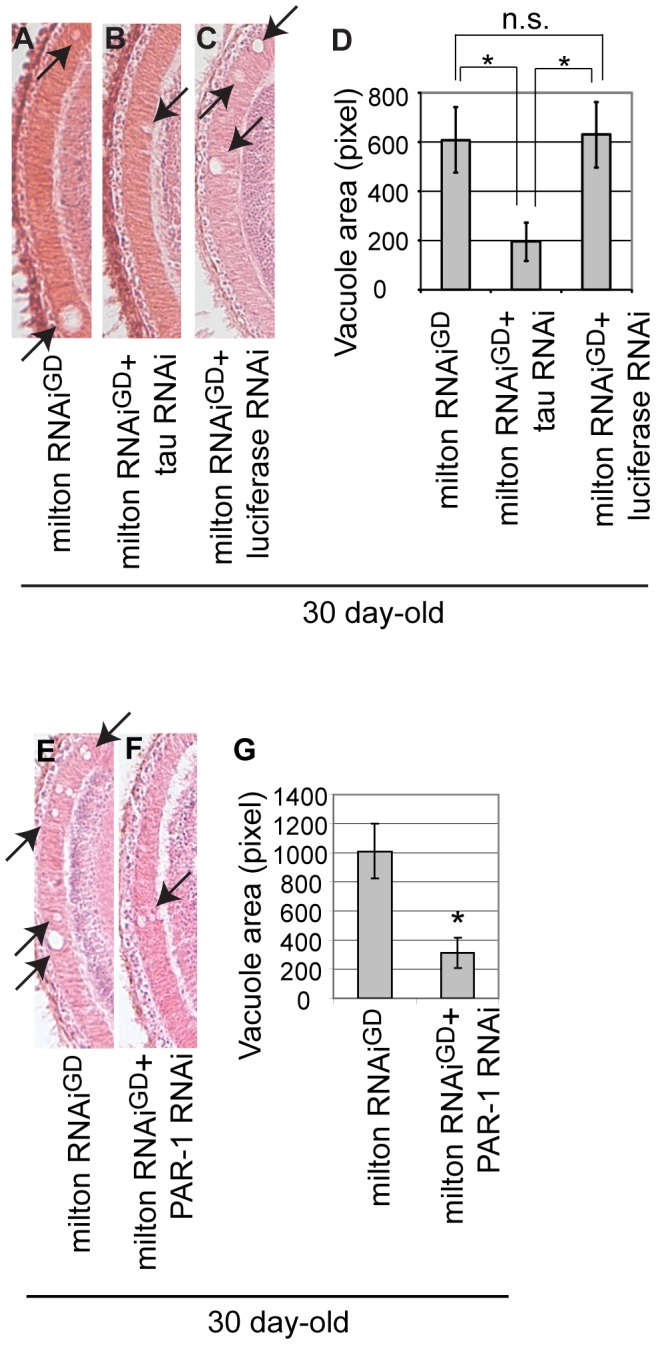
RNAi–mediated knockdown of *Drosophila* tau or PAR-1 suppresses milton knockdown-induced neurodegeneration. (A–D) The lamina of flies expressing milton RNAi alone (A), co-expressing milton RNAi and *Drosophila* tau RNAi (B), and co-expressing milton RNAi and luciferase RNAi (C). (D) Quantification of neurodegeneration (indicated by arrows), mean ± SEM, n = 12. *, p<0.05, Student's t-test. Flies were 30-day-old. (E–G) The lamina of flies expressing milton RNAi alone (E) and co-expressing milton RNAi and PAR-1 RNAi (F). (G) Quantification of neurodegeneration (indicated by arrows), mean ± SEM, n = 10–12. *, p<0.05, Student's t-test. Flies were 30-day-old.

The *Drosophila* tau exhibits a high degree of similarity with the human tau protein and shares a numbers of important features such as the microtubule-binding domains [39] and several phosphorylation sites critical for its functions and toxicity [40]. Overexpression of *Drosophila* tau has been reported to be capable of inducing neuronal dysfunction and neurodegeneration [41]–[43]. We examined whether *Drosophila* tau is involved in neurodegeneration caused by milton knockdown. We found that expression of RNAi targeting *Drosophila* tau reduced tau mRNA levels in the fly brain (Figure S13) and significantly suppressed neurodegeneration in the lamina caused by milton knockdown (Figure 8A and 8B; 8D, quantification). This effect is not due to titration of the effectiveness of RNAi, since the expression of an RNAi against firefly luciferase did not suppress neurodegeneration caused by milton knockdown (Compare Figure 8A and 8C; 8D, quantification). We further examined whether PAR-1 is involved in neurodegeneration caused by milton knockdown. RNAi-mediated knockdown of PAR-1 significantly suppressed neurodegeneration in the lamina of flies expressing milton RNAi (Figure 8E and 8F; 8G, quantification). These results suggest that *Drosophila* endogenous tau and PAR-1 contribute to milton knockdown-induced neurodegeneration and further support the conclusions of this study.

## Discussion

Abnormal phosphorylation and toxicity of tau are thought to play a critical role in the pathogenesis of Alzheimer's disease (AD). Accumulation of amyloid-β peptides is thought to be causative for AD and has been suggested to cause tau abnormality [44]–[53], however, the underlying mechanisms are not clear. A reduction in the number of mitochondria in the axon is observed in the brains of AD patients [3], and we and others previously reported that amyloid-β peptides reduce the number of mitochondria in the axons [3]–[11]. In this study, we examined whether and how loss of axonal mitochondria increases phosphorylation of human tau at AD-related sites and enhances tau toxicity. Our data demonstrate that loss of axonal mitochondria caused by knockdown of milton or Miro increases tau phosphorylation at an AD-related site Ser262 through PAR-1, promotes detachment of tau from microtubules, and enhances tau-mediated neurodegeneration. These results suggest that loss of axonal mitochondria may play an important role in tau phosphorylation and toxicity in the pathogenesis of AD.

It has been reported that, in non-neuronal cultured cells or primary-cultured hippocampal neurons with virus-mediated overexpression of human tau, an excess of microtubule-bound tau blocks microtubule-dependent transport of vesicles and organelle including mitochondria and causes synaptic degeneration [54], [55]. These works have demonstrated that tau phosphorylation at Ser262 by a PAR-1 homolog MARK2 removes tau from the microtubule tracks, which restores microtubule-dependent transport of vesicles and organelle, and rescues accompanied synaptic degeneration [54]. Thus, tau phosphorylation at Ser262 plays a protective role against tau-induced toxicity in their models in which an excess of microtubule-bound tau blocks traffic of vesicles and organelle.

In contrast, this study examined whether and how specific loss of axonal mitochondria promotes tau phosphorylation and toxicity. To address this question *in vivo*, we used a *Drosophila* model of human tau toxicity [22]. In this model, we did not observe severe defects in microtubule-dependent transport under our experimental condition, since mitochondria are present at the synaptic terminals of neurons expressing human tau in the young flies (Figure S2). To chronically deplete mitochondria from the axon, we used knockdown of milton or Miro. The most critical difference between the models used in Thies and Mandelkow [54] and our model is that, in the models of Thies and Mandelkow, mitochondrial transport defects were depending on tau binding to microtubules, while in our model, mitochondria were chronically depleted from the axon by milton knockdown.

Using this model system, we found that milton knockdown significantly enhanced tau-mediated neurodegeneration. Milton knockdown increased the levels of active PAR-1 and tau phosphorylation at Ser262, and promoted detachment of tau from microtubules. If the enhancement of tau toxicity caused by milton knockdown in our model is due to an additive reduction in the number of axonal mitochondria, blocking tau phosphorylation at Ser262, which increases tau binding to microtubules and blocks microtubule-dependent transport, would enhance neurodegeneration. However, our results have shown that blocking tau phosphorylation at Ser262 by PAR-1 knockdown rescues the enhancement of tau-mediated neurodegeneration in the milton knockdown background. These results suggest that the enhancement of tau toxicity in the milton knockdown background is not likely to be due to an additive reduction of axonal transport of mitochondria caused by an excess of microtubule-bound tau. Rather, this study suggests that, when axonal mitochondria are chronically depleted, increased free, microtubule-unbound, Ser262-phosphorylated tau promotes neurodegeneration.

A fine-tuned balance of microtubule-binding of tau is critical for its physiological functions. It has been suggested that both an excess of microtubule-bound tau and an excess of free, microtubule-unbound tau can cause toxicity [56]. Since tau phosphorylation at Ser262 promotes tau detachment from microtubules [32], [33], Ser262 phosphorylation by MARK/PAR-1 plays critical roles under both physiological and pathological conditions. Thus, when axonal distribution of vesicles and organelle are reduced by tau, detachment of tau can play a protective role by temporarily enhancing microtubule-dependent transport [54]. However, our results suggest that, under pathological environments in which axonal mitochondria are chronically depleted, microtubule-unbound, free, Ser262-phosphorylated tau in the axons may become toxic and cause neurodegeneration.

We found that the levels of active PAR-1 are increased by milton knockdown (Figure 6). PAR-1 is activated by various stress stimuli such as high osmolarity and amyloid precursor protein accumulation in *Drosophila* [37]. Our results suggest that loss of axonal mitochondria may trigger a process that increases the levels of active PAR-1. However, the detailed mechanisms by which milton knockdown increases the levels of active PAR-1 require further investigations. PAR-1 activity is regulated by various kinases including LKB1, aPKC, and Death-associated protein kinase (DAPK) [37], [57], [58]. A recent report demonstrated that PAR-1 protein levels were regulated by the *Drosophila* homolog of adducin, a cytoskeletal protein involved in regulating actin filament dynamics [59]. Milton knockdown may act through one or a combination of the mechanisms to increase the level of active PAR-1.

Detachment of tau from microtubules has been suggested to initiate its abnormal metabolism and toxicity of tau in AD pathogenesis [56], however, the underlying mechanisms are not fully understood. This study shows that loss of axonal mitochondria promotes detachment tau from microtubules and enhances tau-mediated neurodegeneration, and tau phosphorylation at AD-related Ser262 by PAR-1 plays a critical role in this process. Our results also suggest that an increase in Ser262-phosphorylated, microtubule-unbound tau may contribute to neurodegeneration under pathological conditions in which axonal mitochondria is depleted. An important question is how free, microtubule-unbound, Ser262-phosphorylated tau causes neurodegeneration under such pathological conditions. Loss of axonal mitochondria would disrupt multiple signaling pathways in the axon, and those changes may further enhance toxicity of tau. Elucidation of such mechanisms will further our understanding of tau-mediated neurodegeneration in the pathogenesis of AD.

In summary, this study highlights a potential role of loss of axonal mitochondria in tau phosphorylation and toxicity in AD pathogenesis. Reductions in the function and number of mitochondria in the axon have also been implicated in several neurodegenerative diseases such as Parkinson's disease, Huntington's disease, and amyotrophic lateral sclerosis [60]. Our study raises an interesting possibility that mitochondrial mislocalization may cause abnormal metabolism and toxicity of other disease-related, aggregation-prone proteins.

## Materials and Methods

### Fly stocks

Transgenic fly lines carrying UAS-luciferase RNAi were established following the method described previously [61]. Target sequences were amplified by PCR from luciferase cDNA using primers (for, 5′-CCGGAATTCGATATGGGCTGAATACAAATCACAGAATCG-3′, rev, 5′-CTAGTCTAGATTCATTAAAACCGGGAGGTAGATGAGATGT-3′ ), and the resulting constructs were subcloned into the pUAST *Drosophila* transformation vector and microinjected into fly embryos of the *w^1118^* genotype. Transgenic fly lines carrying UAS-Miro RNAi were established by microinjecting the Miro RNAi construct (a kind gift from Dr. Barry Dickson (Research Institute of Molecular Pathology, Austria)) into fly embryos of the *w^1118^* genotype. The transgenic fly lines carrying S262A mutant tau was described previously [36]. Other fly stocks are listed in Table S1. Fly genotypes used in each experiment are listed in Table S2.

### Electron microscopy

Probosces were removed from decapitated heads, which were then immersion-fixed overnight in 2.5% glutaraldehyde and 2% paraformaldehyde in 0.1 M sodium cacodylate buffer at 4°C. Samples were post-fixed 1 hr in 1% osmium tetroxide in 0.1 M sodium cacodylate buffer on ice. After washing, samples were stained *en bloc* with 0.5% aqueous uranyl acetate for 1 hr, dehydrated with ethanol and embedded in Epon. Thin-sections (70 nm) of laminas, in which photoreceptor axons were cut longitudinally, were collected on copper grids. The sections were stained with 2% uranyl acetate in 70% ethanol and Reynolds' lead citrate solution. Electron micrographs were obtained with a VELETA CCD Camera (Olympus Soft Imaging Solutions GMBH) mounted on a JEM-1010 electron microscope (Jeol Ltd.).

### Histological analysis

Preparation of paraffin sections, hematoxylin and eosin staining, and analysis of neurodegeneration were described previously [53]. To analyze internal eye structure, heads of female flies were fixed in Bouin's fixative (EMS) for 48 hr at room temperature, incubated 24 hr in 50 mM Tris/150 mM NaCl, and embedded in paraffin. Serial sections (6 µm thickness) through the entire heads were prepared, stained with hematoxylin and eosin (Vector), and examined by bright-field microscopy. Images of the sections that include the lamina were captured with Insight 2 CCD Camera (SPOT), and vacuole area was measured using Image J (NIH). Heads from more than five flies (more than 10 hemispheres) were analyzed for each genotype.

### Western blotting

Twenty fly heads for each genotype were homogenized in SDS-Tris-Glycine sample buffer, and the same amount of the lysate was loaded to each lane of multiple 10% Tris-Glycine gels and transferred to nitrocellulose membrane. The membranes were blocked with 5% milk (Nestle), blotted with the antibodies described below, incubated with appropriate secondary antibody and developed using ECL plus Western Blotting Detection Reagents (GE Healthcare) or imaging with an Odyssey system. One of the membranes was probed with anti-tubulin, and used as the loading control for other blots in each experiment. Anti-tau monoclonal antibody (Tau46, Zymed), and anti-tau pSer262 (Biosource and Calbiochem), phospho-Thr231 (AT180, Thermo and Endogen), anti-HA (Santa Cruz), anti-myc (Millipore), anti-active p44mapk (Promega), anti-tubulin (Sigma), anti-GFP (Clontech) were purchased. Anti-tau pS202 (CP13) was a kind gift from Dr. Peter Davis (Albert Einstein College of Medicine). Anti-PAR-1 pT408 was described previously [37]. The signal intensity was quantified using ImageJ (NIH) or an Odyssey system. Western blots were repeated a minimum of three times with different animals and representative blots are shown. Flies used for Western blotting were 3–5 day-old after eclosion.

### RNA extraction and quantitative real-time PCR analysis

Quantitative real-time PCR analysis was performed as described previously [53]. More than thirty flies for each genotype were collected and frozen. Heads were mechanically isolated, and total RNA was extracted using TRIzol (Invitrogen) according to the manufacturer's protocol with an additional centrifugation step (11,000× g for 10 min) to remove cuticle membranes prior to the addition of chloroform. Total RNA was reverse-transcribed using Superscript II reverse transcriptase (Invitrogen), and the resulting cDNA was used as a template for PCR on a 7500 fast real time PCR system (Applied Biosystems). The average threshold cycle value (Ct) was calculated from five replicates per sample. Expression of genes of interest was standardized relative to actin, rp49 or TBP. Relative expression values were determined by the deltaCt method according to quantitative PCR Analysis User Bulletin (Applied Biosystems). Primers were designed using Primer-Blast (NIH).

milton for 5′-GGCTTCAGGGCCAGGTATCT-3′

milton rev 5′-GCCGAACTTGGCTGACTTTG-3′

Miro for 5′-AAAAGCACCTCATTCTGCGT-3′

Miro rev 5′-CCTCAGGTGAGGAAACGCT-3′

dTau for 5′-AAGCCCGGTGGCGGTGAGAA-3′

dTau rev 5′-GCGCCAGAAGCCGTCATGGA-3′

Actin for 5′-TGCACCGCAAGTGCTTCTAA-3′

Actin rev 5′-TGCTGCACTCCAAACTTCCA-3′

rp49 for 5′-GCTAAGCTGTCGCACAAATG-3′

rp49 rev 5′- GTTCGATCCGTAACCGATGT-3′

TBP for 5′- GCGGCTGTGATTATGCGAAT-3′

TBP rev 5′-AGGGAAACCGAGCTTTTGGA-3′

### Microtubule binding assay

Microtubule binding assay was performed using a previously reported procedure with a modification [62]. Fifty heads from adult flies expressing the human tau protein with the gmr-GAL4 driver were collected and homogenized in 150 µl of Buffer-C+ [50 mM 4-(2-hydroxyethyl)-1-piperazineethanesulfonic acid pH 7.1, 1 mM MgCl2, 1 mM ethylene glycol tetraacetic acid, protease inhibitor cocktail (Roche), and phosphatase inhibitor cocktail (Sigma-Aldrich)] in the presence of taxol 20 µM (Sigma-Aldrich) diluted in dimethylsulfoxide. After centrifugation at 1,000× g for 10 min, aliquot of supernatant was subjected to Western blotting as the “total fraction”. The remaining supernatant was layered onto a 2 volume cushion of buffer-C+ with 50% sucrose. After centrifugation at 100, 000× g for 30 min, the upper fraction containing soluble tubulin was collected as the microtubule-free fraction and the pellet containing microtubule polymers and proteins bound to microtubules was resuspended in 150 µl of SDS-Tris-Glycine sample buffer. Protein concentration in each fraction was measured using the BCA Protein Assay Kit (Pierce). The same amount of protein was loaded to each lane of Tris-Glycine gels and analyzed by western blotting using anti-tau antibody (Tau46, Zymed) or anti-tubulin (Sigma). For quantification, the signal intensity in each lane was quantified with an Odyssey system.

### Statistics

Statistics was done with the JMP software (SAS) with Student's t or Tukey-Kramer HSD. Values are given as mean ± standard deviation or standard error.

## Supporting Information

Figure S1 Expression of human 0N4R wild-type tau causes late-onset, progressive neurodegeneration in the lamina. The lamina expressing tau at 3-day-old (A) or 10-day-old (B), or the lamina of control flies (gmr-GAL4 driver only) at 10-day-old (C). Compare vacuoles indicated by arrows in A (3-day-old) and B (10-day-old). (D) Quantification of the area of vacuoles in the lamina (arrows in A and B), mean ± SEM, n = 14–20. *, p<0.05, Student's t-test. Genotypes are as follows: (tau) +/+;gmr-GAL4/+;UAS-tau/+ and (control) +/+;gmr-GAL4/+;+/+.
(TIF)

Figure S2 Mitochondria are observed in the presynaptic terminals of photoreceptor neurons expressing tau. Transmission electron micrographs of presynaptic terminals in the lamina of flies expressing human tau. Presynaptic terminals are colored green to accentuate the structures. Arrows indicate mitochondria in synaptic terminals. Flies were 3 days-after-eclosion (day-old). Genotype is: +/+;gmr-GAL4/+;UAS-tau/+.
(TIF)

Figure S3 Presynaptic terminals in milton knockdown flies have larger vesicles. Transmission electron micrographs of presynaptic terminals in the lamina of control flies bearing the gmr-GAL4 driver only (control) and flies with milton knockdown (milton RNAi^GD^). Flies were 3 days-after-eclosion (day-old). Genotypes are as follows: (control) +/+;gmr-GAL4/+;+/+ and (milton RNAi^GD^) UAS-milton RNAi^GD^/+;gmr-GAL4/+;+/+.
(TIF)

Figure S4 Milton knockdown causes loss of axonal mitochondria in the photoreceptor neurons expressing human tau in the fly brain. A representative transmission electron micrograph of a presynaptic terminal of a photoreceptor neuron in the lamina of the fly co-expressing human tau and milton RNAi. The presynaptic terminal is colored green to accentuate the structures. Note that mitochondria are not observed (compare to Figure S2). Flies were 3 days-after-eclosion (day-old). Genotype is: UAS-milton RNAi^GD^/+;gmr-GAL4/+;UAS-tau/+.
(TIF)

Figure S5 Milton RNAi^TRiP^ reduces milton mRNA levels and causes mislocalization of mitochondria in the fly brain. (A) Reduction in milton mRNA levels by the expression of milton RNAi^TRiP^ in eyes and neurons. Expression of UAS-luciferase (control) or UAS-milton RNAi^TRiP^ (milton RNAi^TRiP^) was driven by a combination of two drivers, the pan-retinal gmr-GAL4 driver and pan-neuronal elav-GAL4 driver. More than thirty flies for each genotype were collected and frozen. Heads were mechanically isolated, and total RNA was extracted. Milton RNA levels were quantified by qRT-PCR (presented as mean ± SD, n = 5, *p<0.05, Student's t-test). Note that milton RNAi is only expressed in eyes and neurons, while endogenous milton is ubiquitously expressed. Genotypes are as follows: (control) elav-GAL4/Y;gmr-GAL4/+;UAS-luciferase/+ and (milton RNAi^TRiP^) elav-GAL4/Y;gmr-GAL4/+;UAS-milton RNAi^TRiP^/+. (B) Milton RNAi^TRiP^ reduces axonal mitochondrial in the fly brain. (Top) A schematic view of the mushroom body structure, where axons (orange) can be easily identified in the fly brain. (Bottom) Mito-GFP signal in the lobe tips. Ratios relative to control are shown (mean ± SD, n = 6; *, p<0.05, Student's t-test). The mito-GFP signal in the axons was significantly decreased in the milton RNAi^TRiP^ fly brains. Representative images are shown. The method for mito-GFP analysis in the brain is described in Text S1. Genotypes are as follows: (control) elav-GAL4/Y;mito-GFP/+;+/+ and (milton RNAi^TRiP^) elav-GAL4/Y;mito-GFP/+; milton RNAi^TRiP^/+.
(TIF)

Figure S6 Miro RNAi^KK^ reduces axonal mitochondria in the fly brain. Mito-GFP signal in the lobe tips (axons) of the mushroom body structure in the brains of control and Miro RNAi^KK^ flies. Representative images are shown at the top, and the ratio of mito-GFP signal in the lobe tips relative to control are shown at the bottom (mean ± SD, n = 6; *, p<0.05, Student's t-test). Genotypes are as follows: (control) elav-GAL4/Y;mito-GFP/+;+/+ and (Miro RNAi^KK^) elav-GAL4/Y;mito-GFP/UAS- Miro RNAi^KK^;+/+.
(TIF)

Figure S7 Miro RNAi^iai^ causes a reduction in Miro mRNA levels in the fly brain. Expression of UAS-Miro RNAi^iai^ (Miro RNAi^iai^) was driven by the pan-neuronal elav-GAL4 driver. More than thirty flies for control flies (the elav-GAL4 driver only) or Miro RNAi flies were collected and frozen. Heads were mechanically isolated, and total RNA was extracted. Miro mRNA levels were quantified by qRT-PCR (presented as mean ± SD, n = 5, *, p<0. 05, Student's t-test). Note that Miro RNAi^iai^ is only expressed in neurons, while endogenous Miro is ubiquitously expressed. Genotypes are as follows: (control) elav-GAL4/Y;+/+;+/+ and (Miro RNAi) elav-GAL4/Y;+/+;UAS-Miro RNAi^iai^/+.
(TIF)

Figure S8 Tau-mediated neurodegeneration in the lamina is not enhanced by RNAi targeting CG30106, CG4395, CG6064, or CG30340. Quantification of neurodegeneration measured by the area of vacuoles in the lamina, presented as mean ± SEM, n = 10–12. The asterisk indicates significant difference between tau and tau+milton RNAi (p<0.05, Student's t-test). Flies were 3 days-after-eclosion (day-old).
(TIF)

Figure S9 Knockdown of milton or Miro does not enhance tau-mediated reduction in the external eye size. Eyes from flies carrying the pan-retinal gmr-GAL4 driver only (control) (A), expressing human tau alone (tau) (B), co-expressing human tau and milton RNAi (tau+milton RNAi^GD^) (C), co-expressing human tau and Miro RNAi^iai^ (tau+Miro RNAi) (D), or expressing milton RNAi alone (milton RNAi^GD^) (E). Genotypes are as follows: (control) +/+;gmr-GAL4/+;+/+, (tau) +/+;gmr-GAL4/+;UAS-tau/+, (tau+milton RNAi^GD^) UAS-milton RNAi^GD^/+;gmr-GAL4/+;UAS-tau/+, (tau+Miro RNAi) +/+;gmr-GAL4/+;UAS-tau/UAS-Miro RNAi^iai^ and (milton RNAi^GD^) UAS-Milton RNAi^GD^/+;gmr-GAL4/+;+/+.
(TIF)

Figure S10 Expression of S262A tau causes late-onset, progressive neurodegeneration in the lamina. The lamina expressing S262A tau at 3-day-old (A) or 10-day-old (B). Vacuoles in A are indicated by arrows. (C) Quantification of the area of vacuoles in the lamina, mean ± SEM, n = 10–12. *, p<0.05, Student's t-test. Genotype is: +/+;gmr-GAL4/+;UAS-S262Atau/+.
(TIF)

Figure S11 Milton knockdown does not cause non-specific activation of kinases, non-specific accumulation of overexpressed proteins, or an increase in the expression of proteins under the control of GAL4/UAS system. (A) Western blots of eyes from flies expressing HA-tagged p44mapk alone or flies co-expressing p44mapk and milton RNAi^GD^. Blots were probed with anti-phospho-mapk (P-p44mapk) or anti-tubulin. No significant differences were found (mean ± SD, n = 5; p>0.05, Student's t-test). The asterisk indicates non-specific bands. (B) Western blots of eyes from flies expressing HA-tagged p44mapk alone or flies co-expressing p44mapk and milton RNAi^GD^. Blots were probed with anti-HA (p44mapk) or anti-tubulin. No significant differences were found (mean ± SD, n = 5; p>0.05, Student's t-test). (C) Western blots of eyes from flies expressing GFP alone or flies co-expressing GFP and milton RNAi^GD^. Blots were probed with anti-GFP or anti-tubulin. No significant differences were found (mean ± SD, n = 5; p>0.05, Student's t-test). (D) Western blots of eyes from flies expressing myc-tagged APP alone or flies co-expressing APP and milton RNAi^GD^. Blots were probed with anti-myc (APP) or anti-tubulin. No significant differences were found (mean ± SD, n = 5; p>0.05, Student's t-test). All flies were 3 days-after-eclosion (day-old). Genotypes are as follows: (p44mapk) +/+;gmr-GAL4/+;UAS-p44mapk-HA/+, (p44mapk+milton RNAi) UAS-Milton RNAi^GD^/+;gmr-GAL4/+;UAS-p44mapk-HA/+, (GFP) +/+;gmr-GAL4/UAS-GFP;+/+, (GFP+milton RNAi) UAS-Milton RNAi^GD^/+;gmr-GAL4/UAS-GFP;+/+, (APP) +/+;gmr-GAL4/UAS-APP-myc;+/+ and (APP+milton RNAi) UAS-Milton RNAi^GD^/+;gmr-GAL4/UAS-APP-myc;+/+.
(TIF)

Figure S12 Milton knockdown enhances neurodegeneration caused by PAR-1 overexpression. (A–D) PAR-1 overexpression causes late-onset, progressive neurodegeneration in the lamina. The lamina expressing PAR-1 at 3 days-after-eclosion (day-old) (A) or 10-day-old (B), or the lamina of control flies at 10-day-old (C). (D) Quantification of the area of vacuoles in the lamina (arrows in B), mean ± SEM, n = 10–12. *, p<0.05, Student's t-test. (E–H) Milton knockdown enhances PAR-1-induced lamina degeneration. The lamina of flies expressing PAR-1 alone (E), co-expressing PAR-1 and milton RNAi^GD^ (F), and expressing milton RNAi^GD^ alone (G) are shown. (H) Quantification of neurodegeneration (arrows in F), mean ± SEM, n = 10–12. *, significant difference between PAR-1 and PAR-1+milton RNAi^GD^ (p<0.05, Student's t-test). Genotypes are as follows: (control) +/+;gmr-GAL4/+;+/+, (PAR-1) +/+;gmr-GAL4/+;UAS-PAR-1-myc/+, (PAR-1+milton RNAi^GD^) UAS-Milton RNAi^GD^/+;gmr-GAL4/+;UAS-PAR-1-myc/+ and (milton RNAi^GD^) UAS-Milton RNAi^GD^/+;gmr-GAL4/+;+/+.
(TIF)

Figure S13 Tau RNAi causes a reduction in tau mRNA levels in the fly brain. Expression of UAS-luciferase (control) or UAS-tau RNAi (tau RNAi) was driven by a combination of two drivers, the pan-retinal gmr-GAL4 driver and pan-neuronal elav-GAL4 driver. More than thirty flies for each genotype were collected and frozen. Heads were mechanically isolated, and total RNA was extracted. Tau mRNA levels were quantified by qRT-PCR (presented as mean ± SD, n = 5, *, p<0.05, Student's t-test). Genotypes are as follows: (control) elav-GAL4/Y;gmr-GAL4/+;UAS-luciferase/+, and (tau RNAi) elav-GAL4/Y;gmr-GAL4/+;UAS-tau RNAi/+.
(TIF)

Table S1 Fly stocks.
(DOC)

Table S2 Genotypes of the flies that were used in each experiment.
(DOC)

Text S1 Materials and methods for mito-GFP analysis in fly brains.
(DOC)
