# Supplementary figures and images for: Loss of Axonal Mitochondria Promotes Tau-Mediated Neurodegeneration and Alzheimer's Disease–Related Tau Phosphorylation Via PAR-1
Source: PLoS Genet. 2012 Aug 30;8(8):e1002918. doi: 10.1371/journal.pgen.1002918 (PMC3431335; doi:10.1371/journal.pgen.1002918)

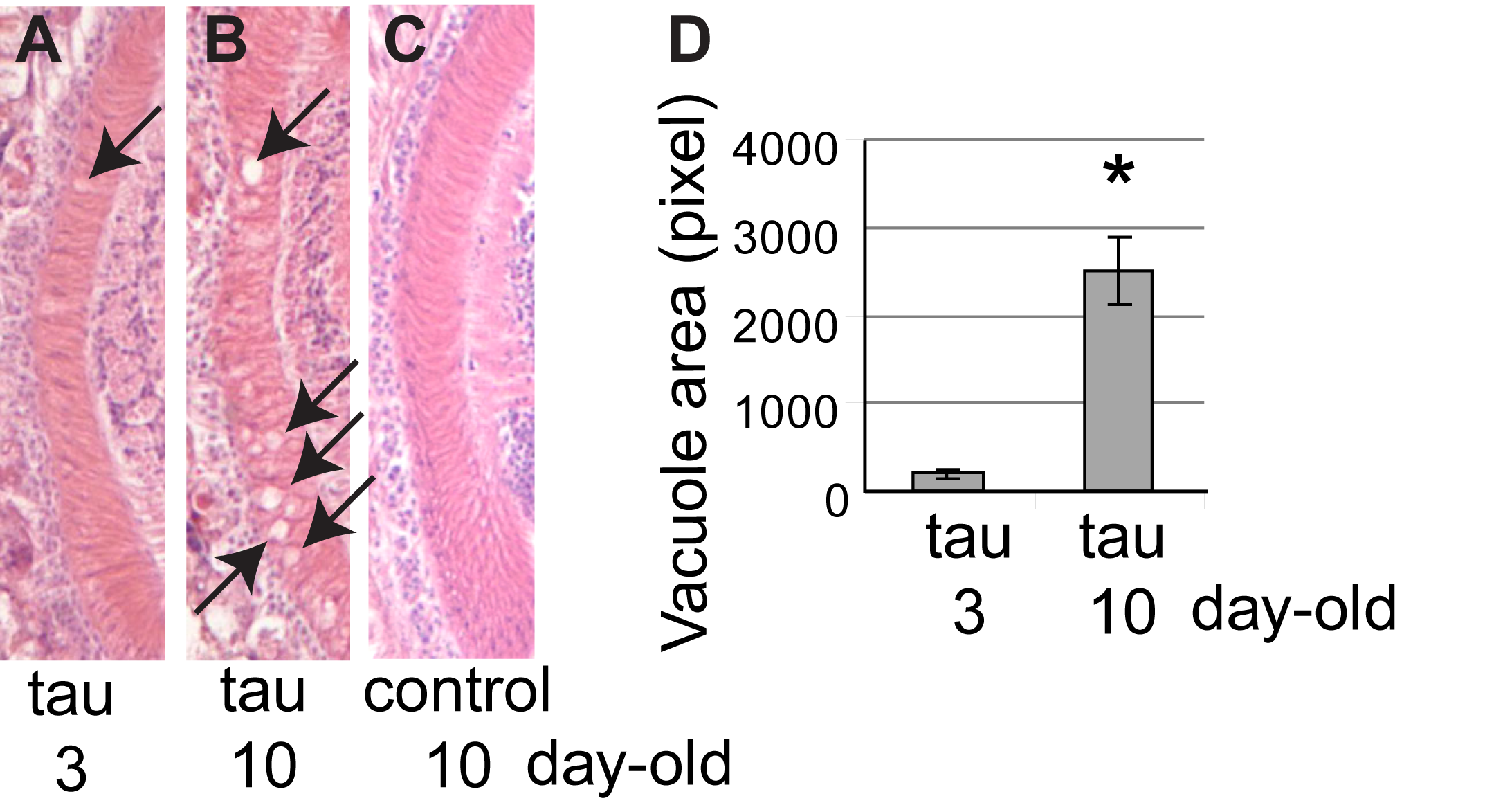

Supplement: Figure S1 — Expression of human 0N4R wild-type tau causes late-onset, progressive neurodegeneration in the lamina. The lamina expressing tau at 3-day-old (A) or 10-day-old (B), or the lamina of control flies (gmr-GAL4 driver only) at 10-day-old (C). Compare vacuoles indicated by arrows in A (3-day-old) and B (10-day-old). (D) Quantification of the area of vacuoles in the lamina (arrows in A and B), mean ± SEM, n = 14–20. *, p<0.05, Student's t-test. Genotypes are as follows: (tau) +/+;gmr-GAL4/+;UAS-tau/+ and (control) +/+;gmr-GAL4/+;+/+. (TIF) [file pgen.1002918.s001.tif]

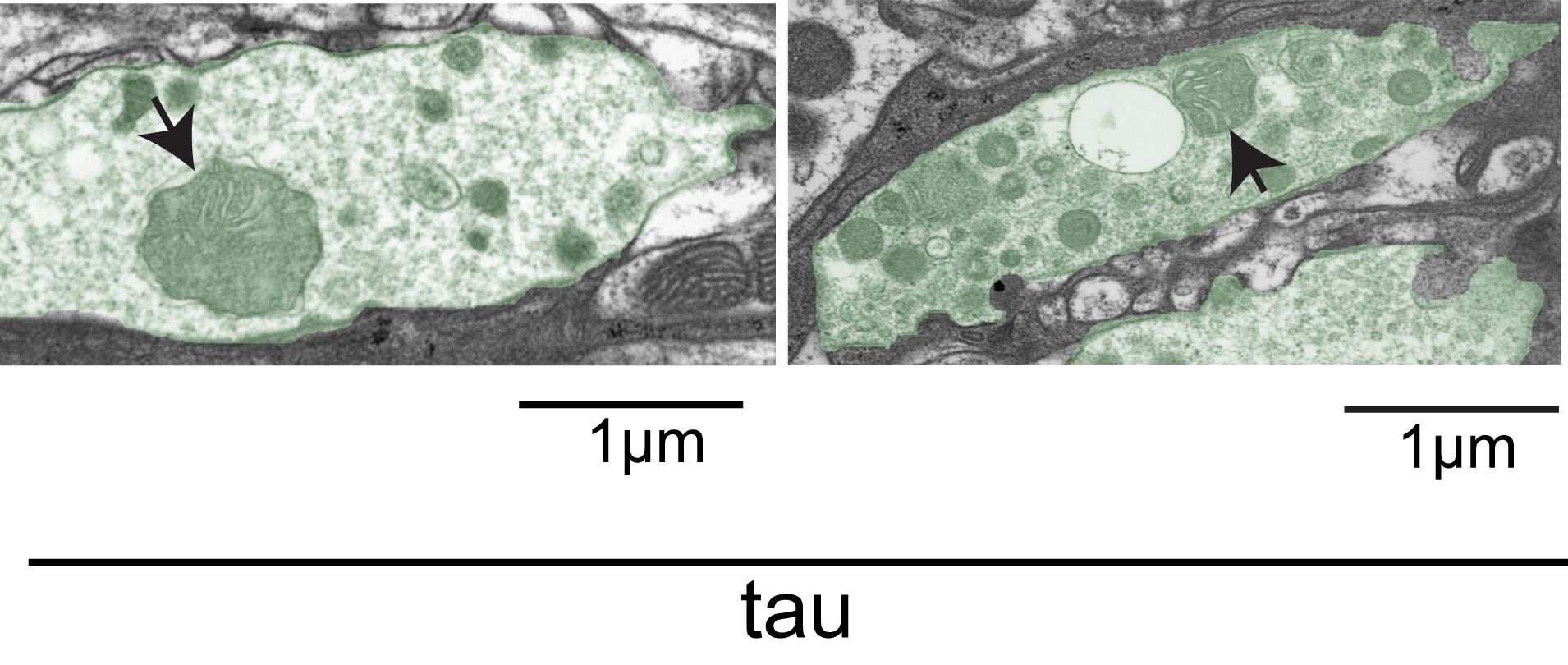

Supplement: Figure S2 — Mitochondria are observed in the presynaptic terminals of photoreceptor neurons expressing tau. Transmission electron micrographs of presynaptic terminals in the lamina of flies expressing human tau. Presynaptic terminals are colored green to accentuate the structures. Arrows indicate mitochondria in synaptic terminals. Flies were 3 days-after-eclosion (day-old). Genotype is: +/+;gmr-GAL4/+;UAS-tau/+. (TIF) [file pgen.1002918.s002.tif]

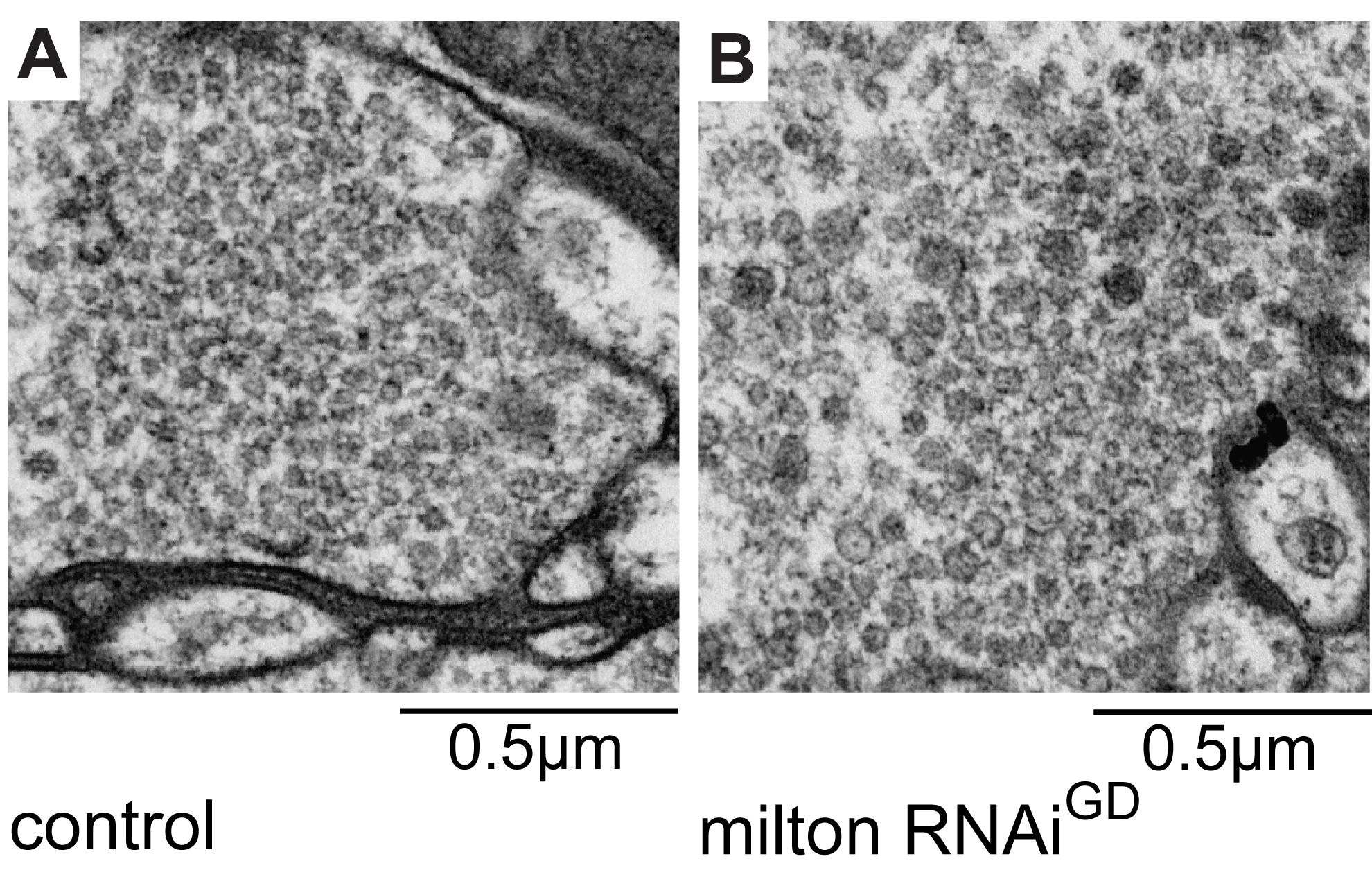

Supplement: Figure S3 — Presynaptic terminals in milton knockdown flies have larger vesicles. Transmission electron micrographs of presynaptic terminals in the lamina of control flies bearing the gmr-GAL4 driver only (control) and flies with milton knockdown (milton RNAiGD). Flies were 3 days-after-eclosion (day-old). Genotypes are as follows: (control) +/+;gmr-GAL4/+;+/+ and (milton RNAiGD) UAS-milton RNAiGD/+;gmr-GAL4/+;+/+. (TIF) [file pgen.1002918.s003.tif]

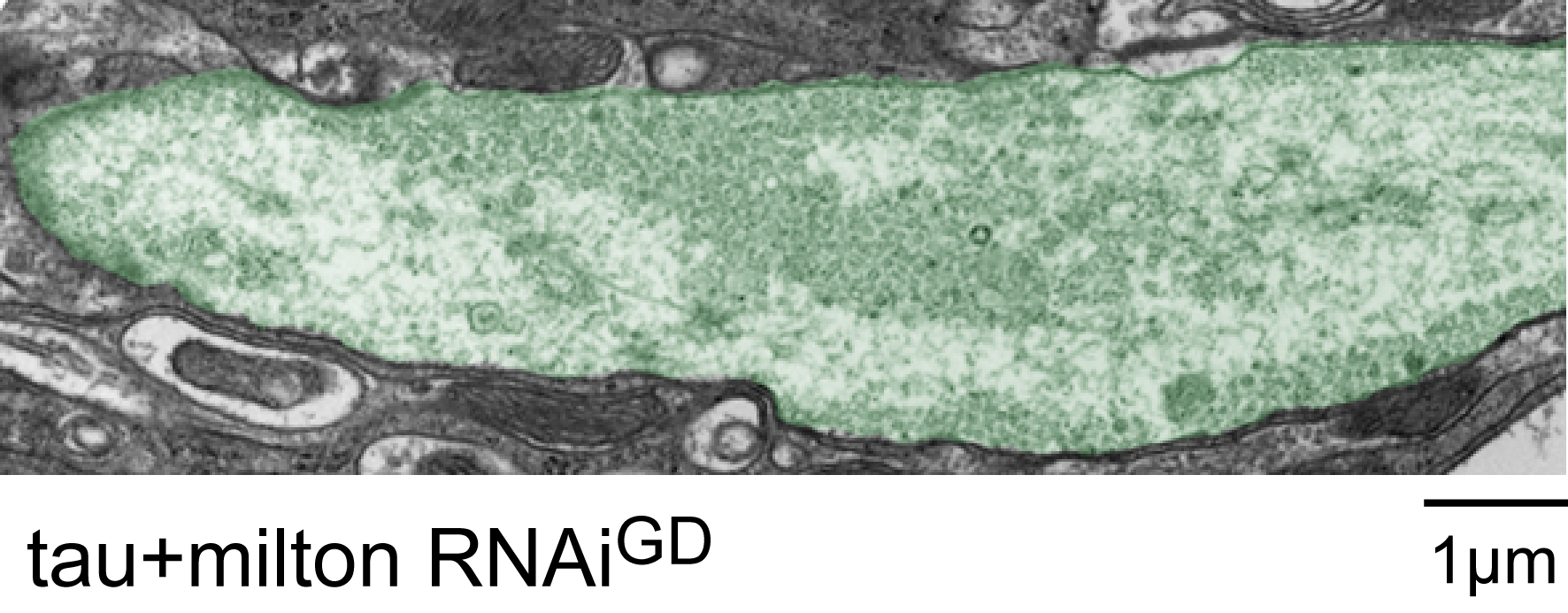

Supplement: Figure S4 — Milton knockdown causes loss of axonal mitochondria in the photoreceptor neurons expressing human tau in the fly brain. A representative transmission electron micrograph of a presynaptic terminal of a photoreceptor neuron in the lamina of the fly co-expressing human tau and milton RNAi. The presynaptic terminal is colored green to accentuate the structures. Note that mitochondria are not observed (compare to Figure S2). Flies were 3 days-after-eclosion (day-old). Genotype is: UAS-milton RNAiGD/+;gmr-GAL4/+;UAS-tau/+. (TIF) [file pgen.1002918.s004.tif]

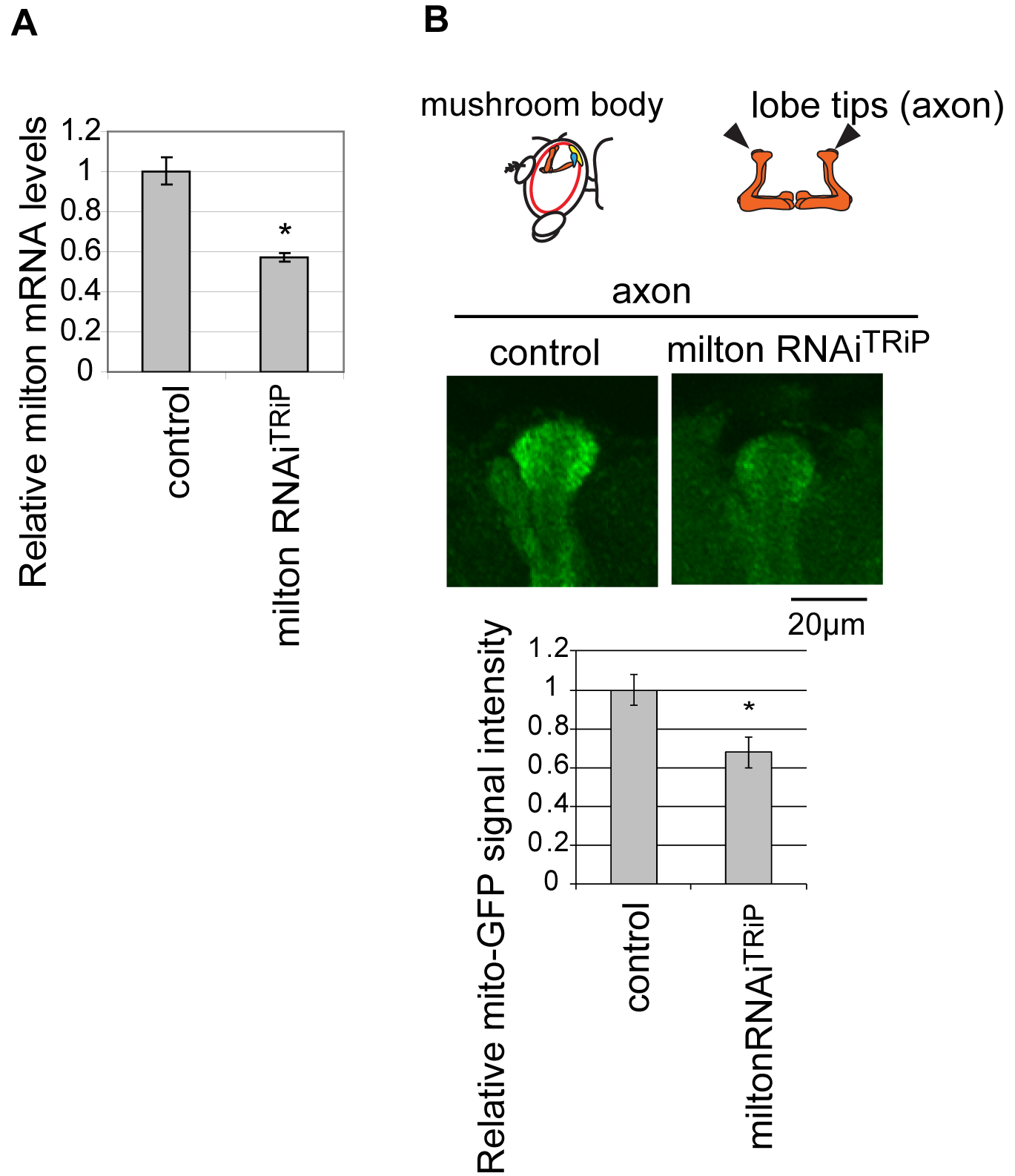

Supplement: Figure S5 — Milton RNAiTRiP reduces milton mRNA levels and causes mislocalization of mitochondria in the fly brain. (A) Reduction in milton mRNA levels by the expression of milton RNAiTRiP in eyes and neurons. Expression of UAS-luciferase (control) or UAS-milton RNAiTRiP (milton RNAiTRiP) was driven by a combination of two drivers, the pan-retinal gmr-GAL4 driver and pan-neuronal elav-GAL4 driver. More than thirty flies for each genotype were collected and frozen. Heads were mechanically isolated, and total RNA was extracted. Milton RNA levels were quantified by qRT-PCR (presented as mean ± SD, n = 5, *p<0.05, Student's t-test). Note that milton RNAi is only expressed in eyes and neurons, while endogenous milton is ubiquitously expressed. Genotypes are as follows: (control) elav-GAL4/Y;gmr-GAL4/+;UAS-luciferase/+ and (milton RNAiTRiP) elav-GAL4/Y;gmr-GAL4/+;UAS-milton RNAiTRiP/+. (B) Milton RNAiTRiP reduces axonal mitochondrial in the fly brain. (Top) A schematic view of the mushroom body structure, where axons (orange) can be easily identified in the fly brain. (Bottom) Mito-GFP signal in the lobe tips. Ratios relative to control are shown (mean ± SD, n = 6; *, p<0.05, Student's t-test). The mito-GFP signal in the axons was significantly decreased in the milton RNAiTRiP fly brains. Representative images are shown. The method for mito-GFP analysis in the brain is described in Text S1. Genotypes are as follows: (control) elav-GAL4/Y;mito-GFP/+;+/+ and (milton RNAiTRiP) elav-GAL4/Y;mito-GFP/+; milton RNAiTRiP/+. (TIF) [file pgen.1002918.s005.tif]

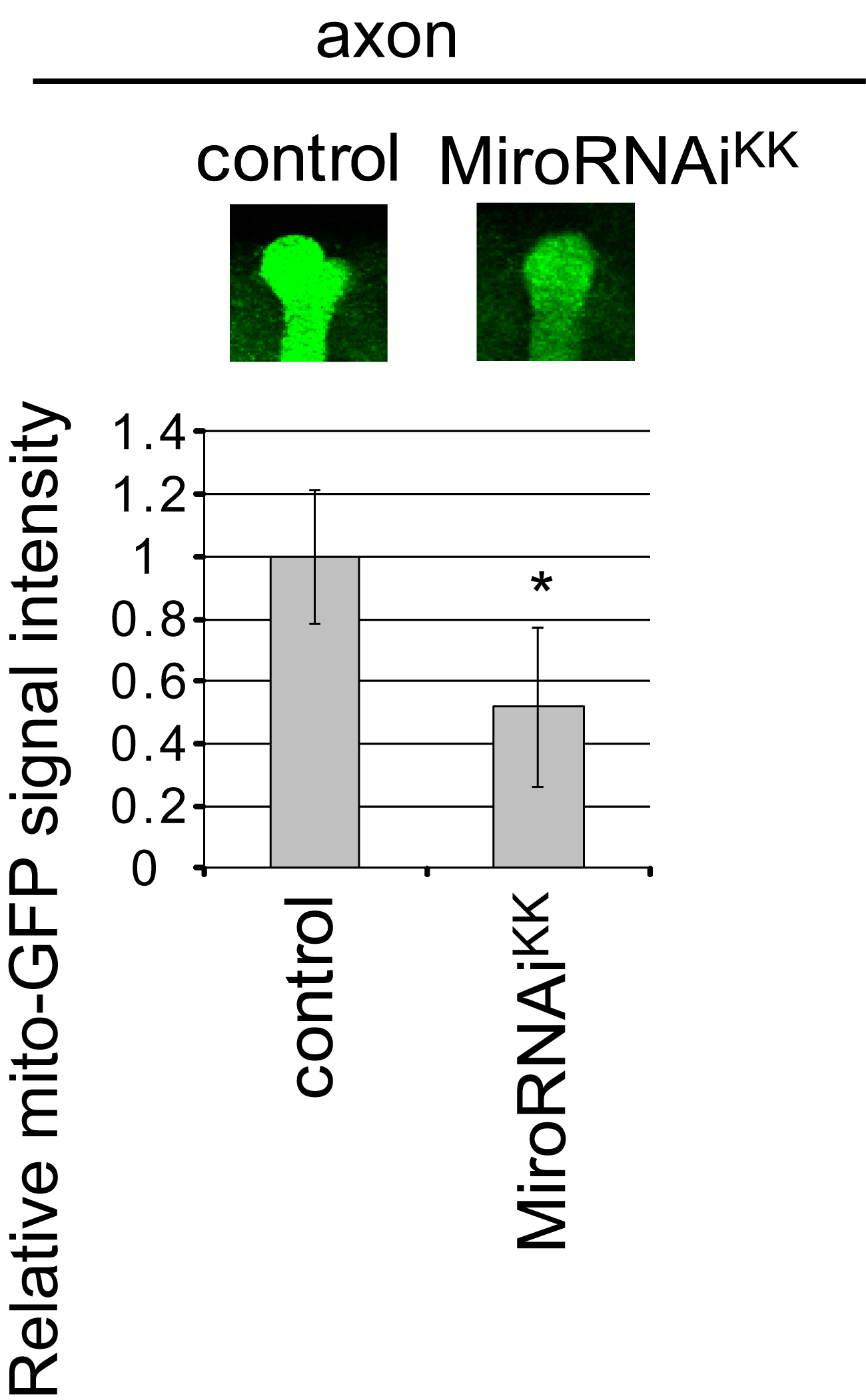

Supplement: Figure S6 — Miro RNAiKK reduces axonal mitochondria in the fly brain. Mito-GFP signal in the lobe tips (axons) of the mushroom body structure in the brains of control and Miro RNAiKK flies. Representative images are shown at the top, and the ratio of mito-GFP signal in the lobe tips relative to control are shown at the bottom (mean ± SD, n = 6; *, p<0.05, Student's t-test). Genotypes are as follows: (control) elav-GAL4/Y;mito-GFP/+;+/+ and (Miro RNAiKK) elav-GAL4/Y;mito-GFP/UAS- Miro RNAiKK;+/+. (TIF) [file pgen.1002918.s006.tif]

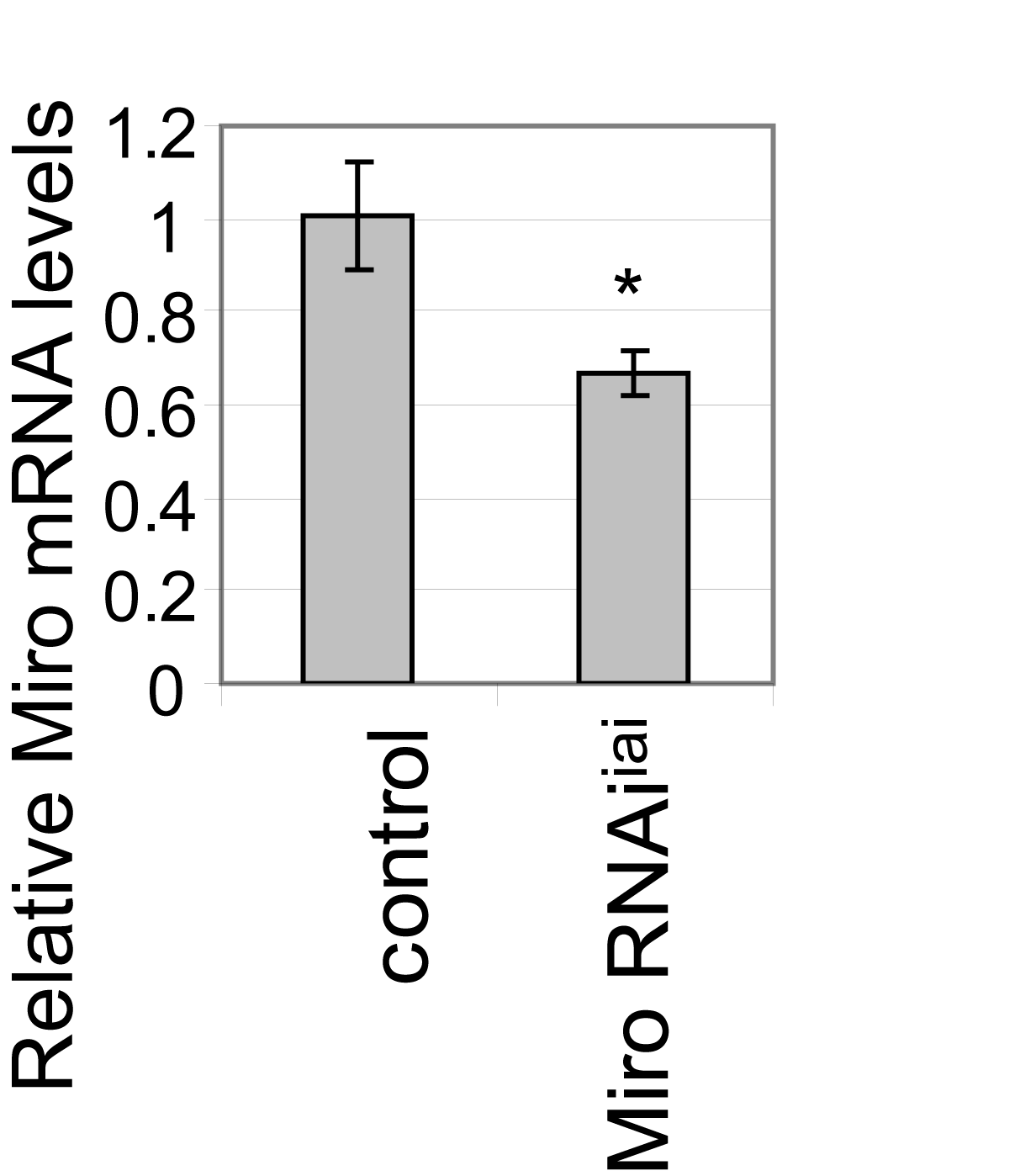

Supplement: Figure S7 — Miro RNAiiai causes a reduction in Miro mRNA levels in the fly brain. Expression of UAS-Miro RNAiiai (Miro RNAiiai) was driven by the pan-neuronal elav-GAL4 driver. More than thirty flies for control flies (the elav-GAL4 driver only) or Miro RNAi flies were collected and frozen. Heads were mechanically isolated, and total RNA was extracted. Miro mRNA levels were quantified by qRT-PCR (presented as mean ± SD, n = 5, *, p<0. 05, Student's t-test). Note that Miro RNAiiai is only expressed in neurons, while endogenous Miro is ubiquitously expressed. Genotypes are as follows: (control) elav-GAL4/Y;+/+;+/+ and (Miro RNAi) elav-GAL4/Y;+/+;UAS-Miro RNAiiai/+. (TIF) [file pgen.1002918.s007.tif]

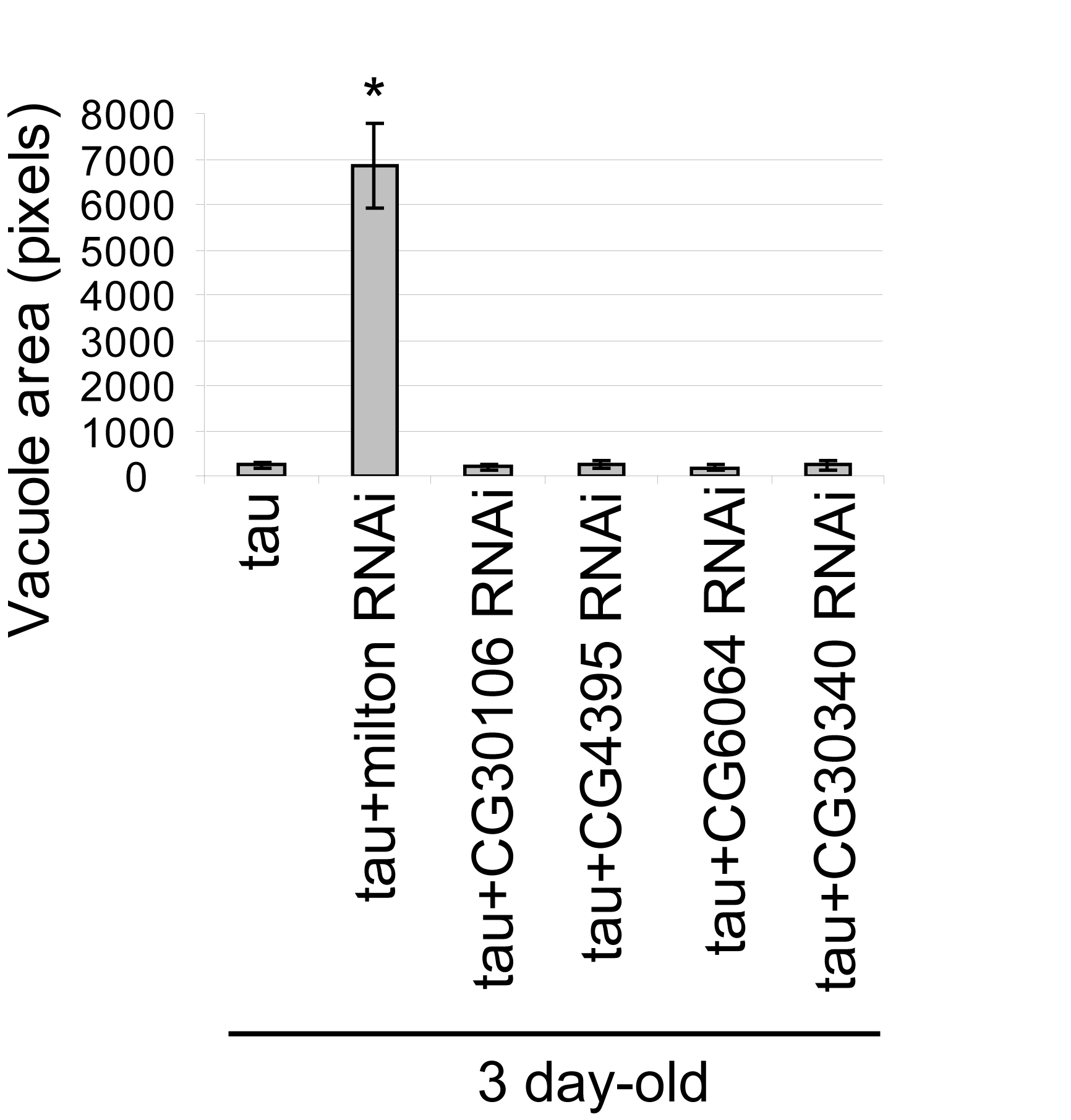

Supplement: Figure S8 — Tau-mediated neurodegeneration in the lamina is not enhanced by RNAi targeting CG30106, CG4395, CG6064, or CG30340. Quantification of neurodegeneration measured by the area of vacuoles in the lamina, presented as mean ± SEM, n = 10–12. The asterisk indicates significant difference between tau and tau+milton RNAi (p<0.05, Student's t-test). Flies were 3 days-after-eclosion (day-old). (TIF) [file pgen.1002918.s008.tif]

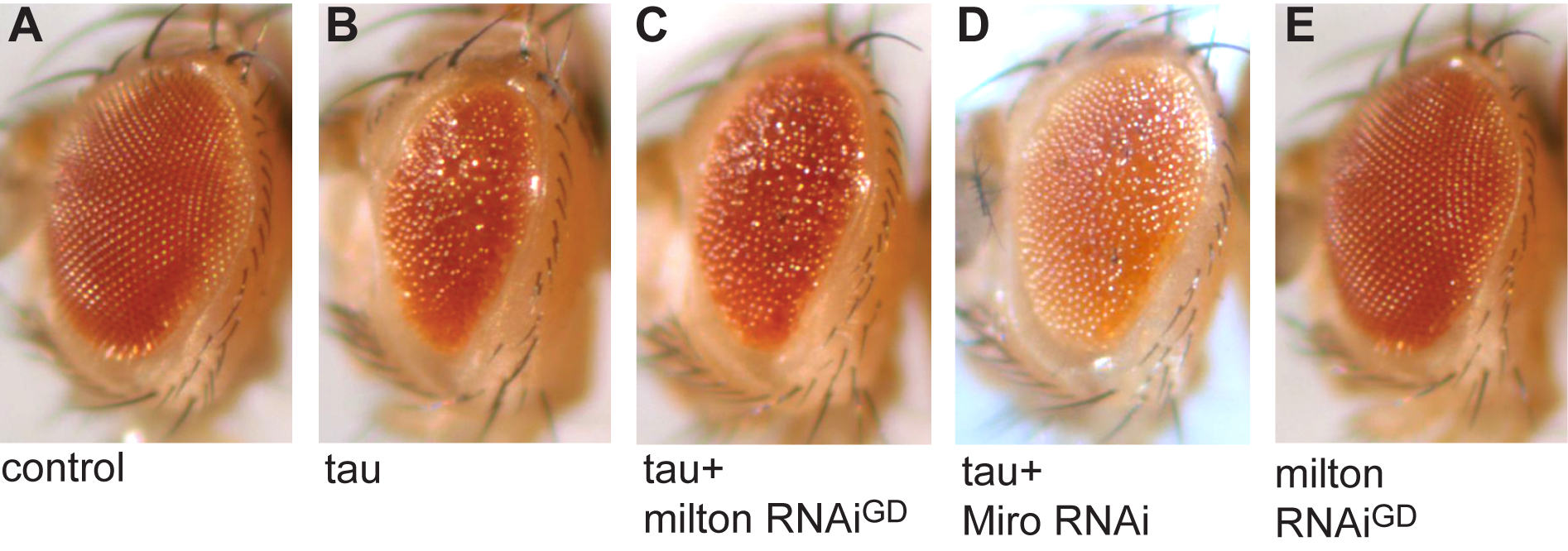

Supplement: Figure S9 — Knockdown of milton or Miro does not enhance tau-mediated reduction in the external eye size. Eyes from flies carrying the pan-retinal gmr-GAL4 driver only (control) (A), expressing human tau alone (tau) (B), co-expressing human tau and milton RNAi (tau+milton RNAiGD) (C), co-expressing human tau and Miro RNAiiai (tau+Miro RNAi) (D), or expressing milton RNAi alone (milton RNAiGD) (E). Genotypes are as follows: (control) +/+;gmr-GAL4/+;+/+, (tau) +/+;gmr-GAL4/+;UAS-tau/+, (tau+milton RNAiGD) UAS-milton RNAiGD/+;gmr-GAL4/+;UAS-tau/+, (tau+Miro RNAi) +/+;gmr-GAL4/+;UAS-tau/UAS-Miro RNAiiai and (milton RNAiGD) UAS-Milton RNAiGD/+;gmr-GAL4/+;+/+. (TIF) [file pgen.1002918.s009.tif]

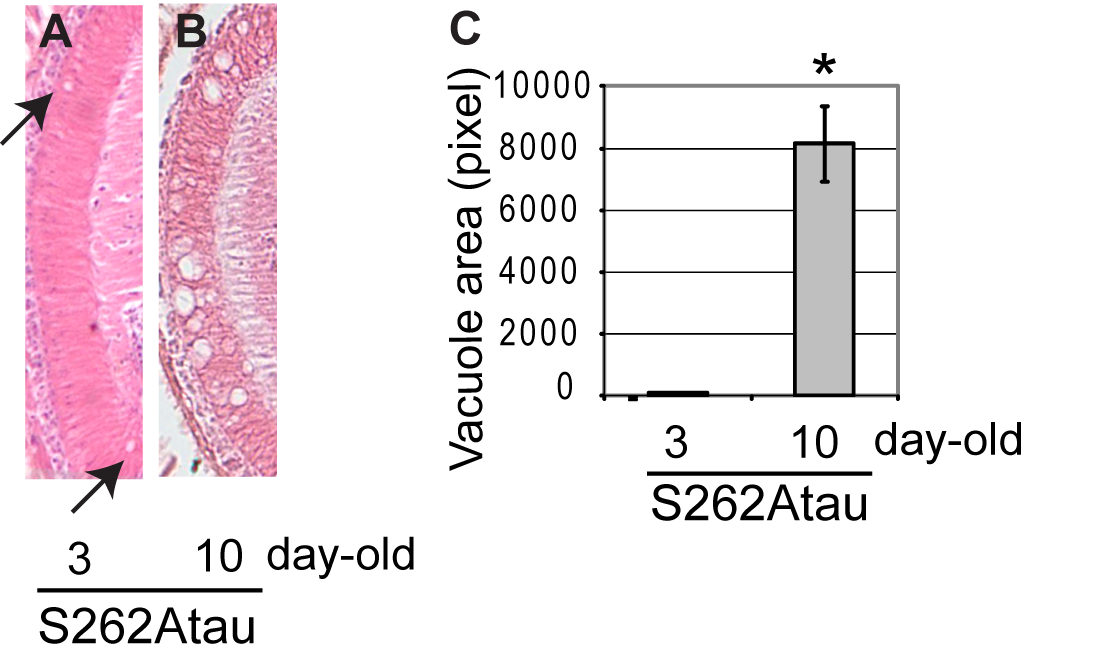

Supplement: Figure S10 — Expression of S262A tau causes late-onset, progressive neurodegeneration in the lamina. The lamina expressing S262A tau at 3-day-old (A) or 10-day-old (B). Vacuoles in A are indicated by arrows. (C) Quantification of the area of vacuoles in the lamina, mean ± SEM, n = 10–12. *, p<0.05, Student's t-test. Genotype is: +/+;gmr-GAL4/+;UAS-S262Atau/+. (TIF) [file pgen.1002918.s010.tif]

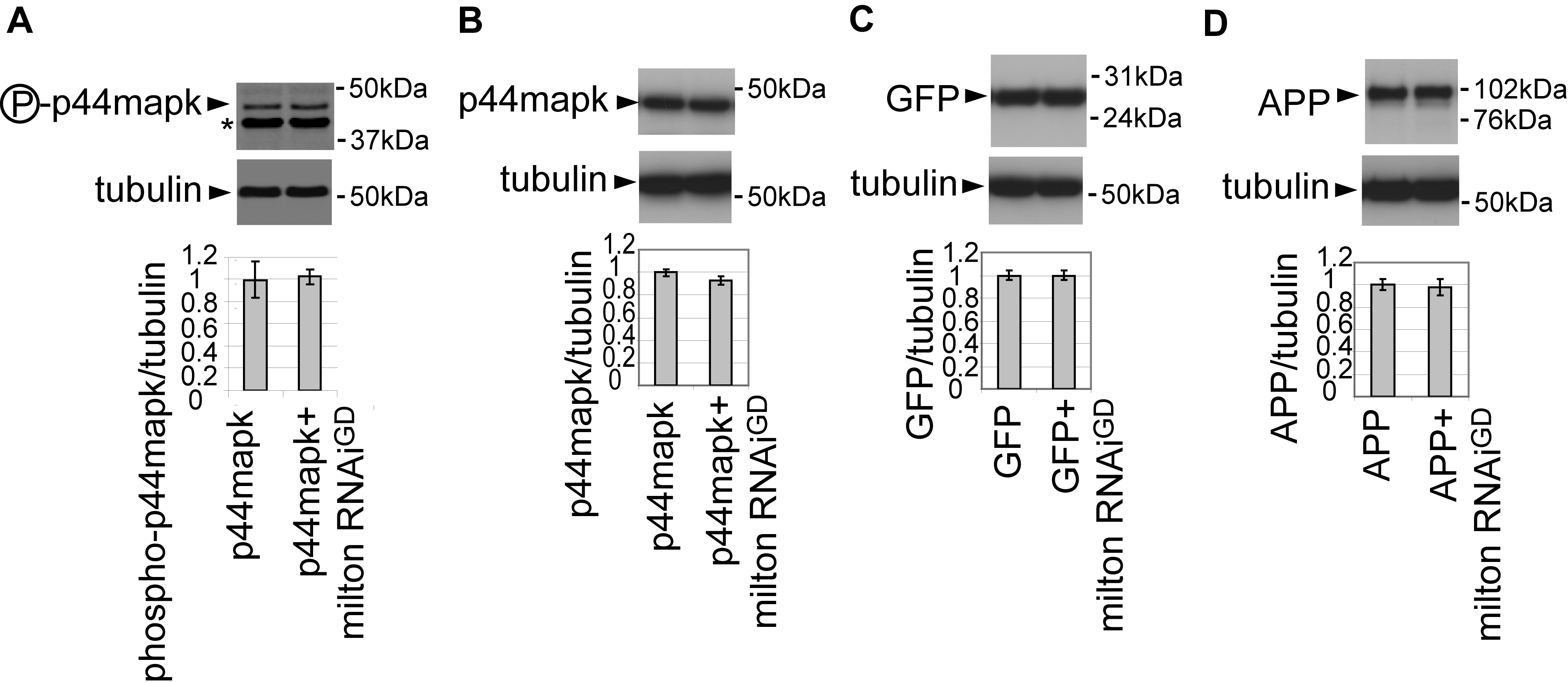

Supplement: Figure S11 — Milton knockdown does not cause non-specific activation of kinases, non-specific accumulation of overexpressed proteins, or an increase in the expression of proteins under the control of GAL4/UAS system. (A) Western blots of eyes from flies expressing HA-tagged p44mapk alone or flies co-expressing p44mapk and milton RNAiGD. Blots were probed with anti-phospho-mapk (P-p44mapk) or anti-tubulin. No significant differences were found (mean ± SD, n = 5; p>0.05, Student's t-test). The asterisk indicates non-specific bands. (B) Western blots of eyes from flies expressing HA-tagged p44mapk alone or flies co-expressing p44mapk and milton RNAiGD. Blots were probed with anti-HA (p44mapk) or anti-tubulin. No significant differences were found (mean ± SD, n = 5; p>0.05, Student's t-test). (C) Western blots of eyes from flies expressing GFP alone or flies co-expressing GFP and milton RNAiGD. Blots were probed with anti-GFP or anti-tubulin. No significant differences were found (mean ± SD, n = 5; p>0.05, Student's t-test). (D) Western blots of eyes from flies expressing myc-tagged APP alone or flies co-expressing APP and milton RNAiGD. Blots were probed with anti-myc (APP) or anti-tubulin. No significant differences were found (mean ± SD, n = 5; p>0.05, Student's t-test). All flies were 3 days-after-eclosion (day-old). Genotypes are as follows: (p44mapk) +/+;gmr-GAL4/+;UAS-p44mapk-HA/+, (p44mapk+milton RNAi) UAS-Milton RNAiGD/+;gmr-GAL4/+;UAS-p44mapk-HA/+, (GFP) +/+;gmr-GAL4/UAS-GFP;+/+, (GFP+milton RNAi) UAS-Milton RNAiGD/+;gmr-GAL4/UAS-GFP;+/+, (APP) +/+;gmr-GAL4/UAS-APP-myc;+/+ and (APP+milton RNAi) UAS-Milton RNAiGD/+;gmr-GAL4/UAS-APP-myc;+/+. (TIF) [file pgen.1002918.s011.tif]

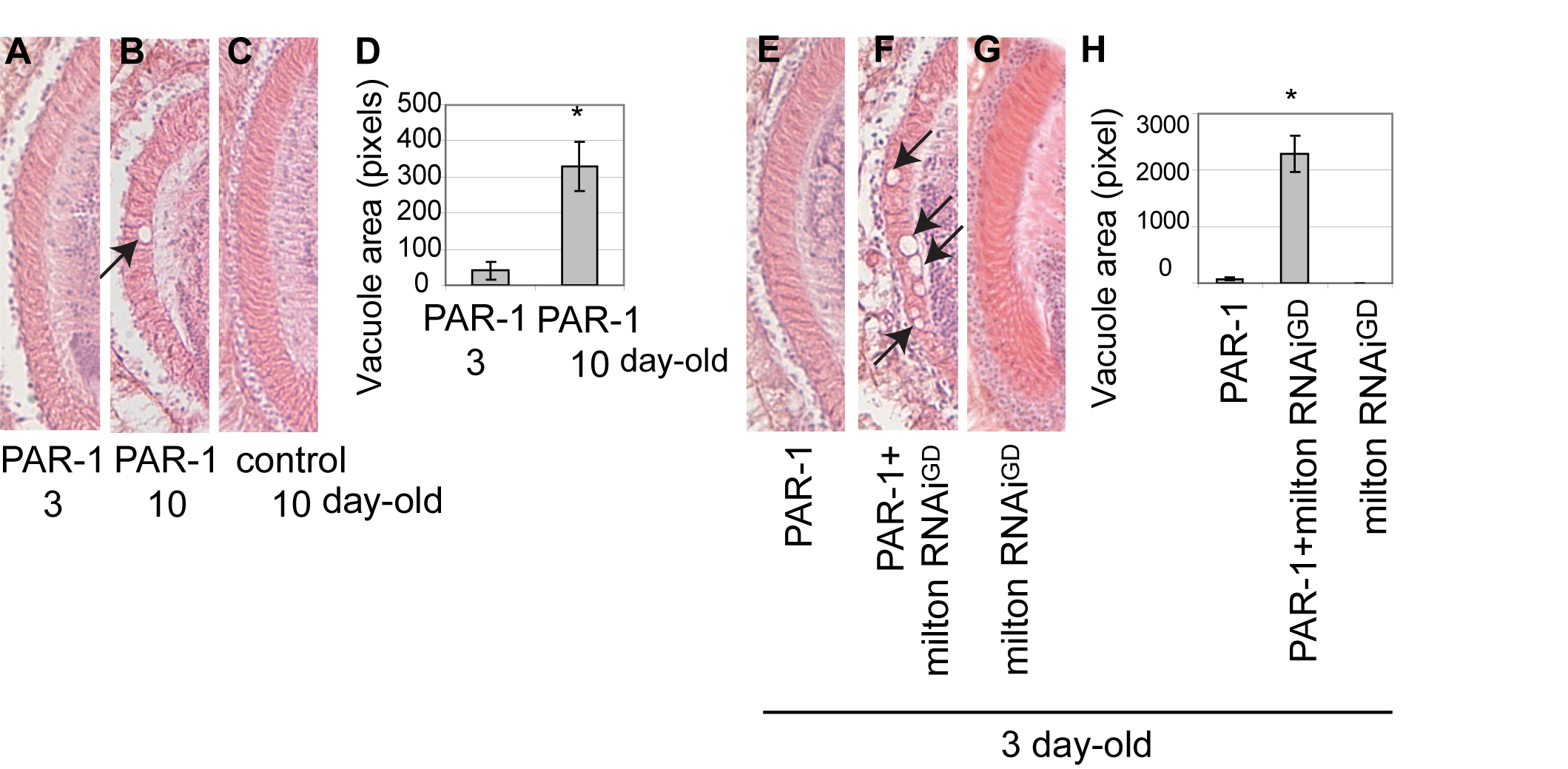

Supplement: Figure S12 — Milton knockdown enhances neurodegeneration caused by PAR-1 overexpression. (A–D) PAR-1 overexpression causes late-onset, progressive neurodegeneration in the lamina. The lamina expressing PAR-1 at 3 days-after-eclosion (day-old) (A) or 10-day-old (B), or the lamina of control flies at 10-day-old (C). (D) Quantification of the area of vacuoles in the lamina (arrows in B), mean ± SEM, n = 10–12. *, p<0.05, Student's t-test. (E–H) Milton knockdown enhances PAR-1-induced lamina degeneration. The lamina of flies expressing PAR-1 alone (E), co-expressing PAR-1 and milton RNAiGD (F), and expressing milton RNAiGD alone (G) are shown. (H) Quantification of neurodegeneration (arrows in F), mean ± SEM, n = 10–12. *, significant difference between PAR-1 and PAR-1+milton RNAiGD (p<0.05, Student's t-test). Genotypes are as follows: (control) +/+;gmr-GAL4/+;+/+, (PAR-1) +/+;gmr-GAL4/+;UAS-PAR-1-myc/+, (PAR-1+milton RNAiGD) UAS-Milton RNAiGD/+;gmr-GAL4/+;UAS-PAR-1-myc/+ and (milton RNAiGD) UAS-Milton RNAiGD/+;gmr-GAL4/+;+/+. (TIF) [file pgen.1002918.s012.tif]

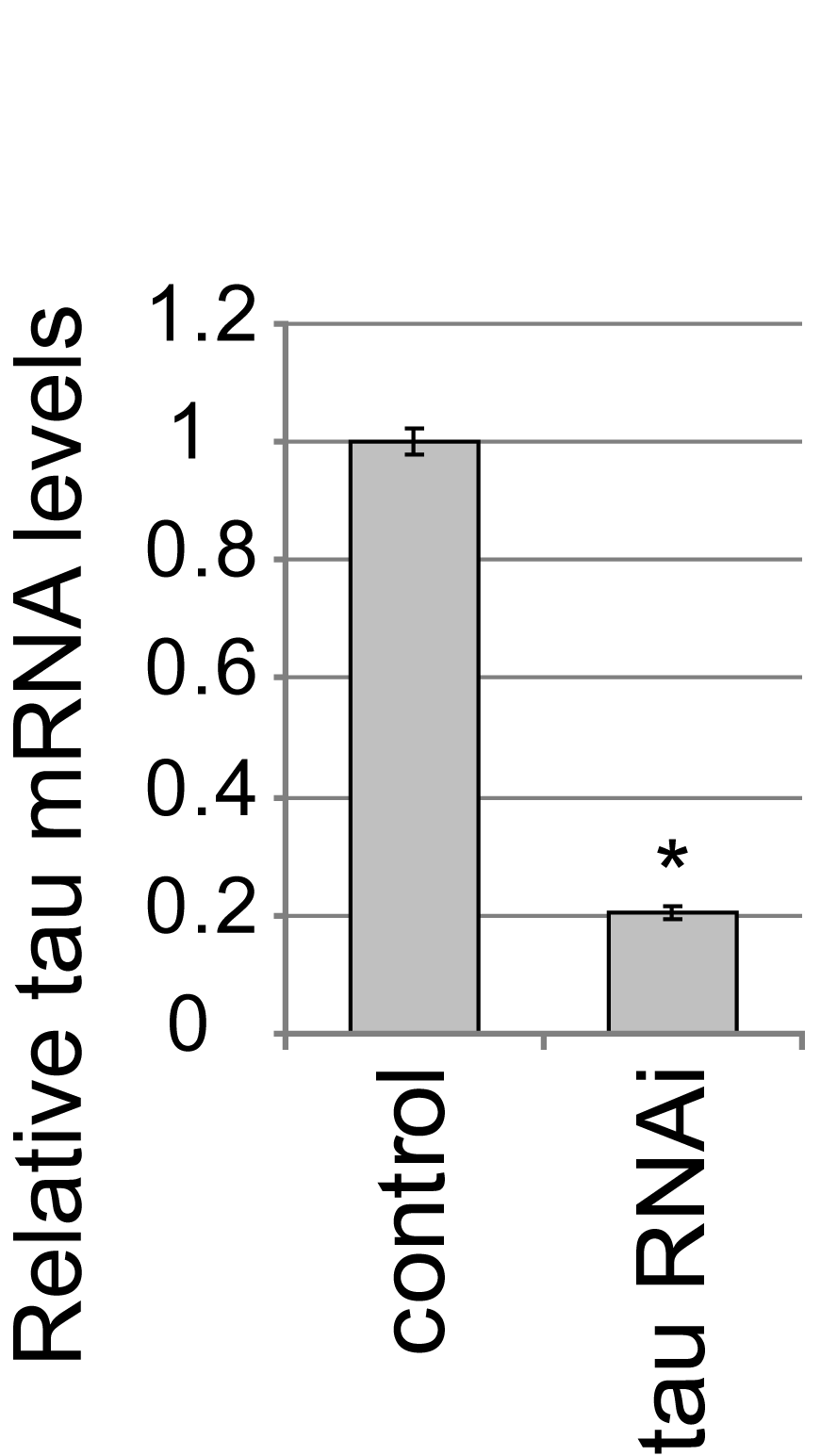

Supplement: Figure S13 — Tau RNAi causes a reduction in tau mRNA levels in the fly brain. Expression of UAS-luciferase (control) or UAS-tau RNAi (tau RNAi) was driven by a combination of two drivers, the pan-retinal gmr-GAL4 driver and pan-neuronal elav-GAL4 driver. More than thirty flies for each genotype were collected and frozen. Heads were mechanically isolated, and total RNA was extracted. Tau mRNA levels were quantified by qRT-PCR (presented as mean ± SD, n = 5, *, p<0.05, Student's t-test). Genotypes are as follows: (control) elav-GAL4/Y;gmr-GAL4/+;UAS-luciferase/+, and (tau RNAi) elav-GAL4/Y;gmr-GAL4/+;UAS-tau RNAi/+. (TIF) [file pgen.1002918.s013.tif]
