# Supplementary material for: Loss of Axonal Mitochondria Promotes Tau-Mediated Neurodegeneration and Alzheimer's Disease–Related Tau Phosphorylation Via PAR-1
Source: PLoS Genet. 2012 Aug 30;8(8):e1002918. doi: 10.1371/journal.pgen.1002918 (PMC3431335; doi:10.1371/journal.pgen.1002918)
Supplement: Table S1 — Fly stocks. (DOC) [file pgen.1002918.s014.doc]

| **Transgenic fly** | **From** | **Reference** |
| --- | --- | --- |
| UAS-human 0N4R tau, which has four tubulin-binding domains (R) and no N-terminal insert (N) | Dr. Mel Feany (Harvard Medical School) | [1] |
| gmr-GAL4 | the Bloomington *Drosophila* Stock Center | [2] |
| UAS-milton RNAi^GD^ (v41508) | Vienna *Drosophila* RNAi Center | [3,4] |
| UAS-milton RNAi ^TRiP^ (JF03022) | the Bloomington *Drosophila* Stock Center | TRiP at Harvard Medical School |
| UAS-Miro RNAi^KK^ (v106683) | Vienna *Drosophila* RNAi Center | [3,5] |
| UAS-Miro RNAi^iai^ |  | this study |
| UAS-luciferase RNAi |  | this study |
| UAS-CG30106 RNAi (v1678) | Vienna *Drosophila* RNAi Center | [3] |
| UAS-CG4395 RNAi (v7223) | Vienna *Drosophila* RNAi Center | [3] |
| UAS-CG6064 RNAi (v27545) | Vienna *Drosophila* RNAi Center | [3] |
| UAS-CG30340 RNAi (v7387) | Vienna *Drosophila* RNAi Center | [3] |
| UAS-PAR-1 RNAi | Dr. Jocelyn McDonald (Cleveland Clinic) | [6] |
| UAS-tau S262A |  | [7] |
| UAS-PAR-1-myc |  | [8] |
| UAS-PAR-1 T408A-myc |  | [8] |
| UAS-p44mapk-HA | Dr. Jong Kyeong Chung (Korea Advanced Institute of Science and Technology) | [9] |
| UAS-APP-myc | the Bloomington *Drosophila* Stock Center | [10] |
| UAS-GFP | the Bloomington *Drosophila* Stock Center |  |
| elav-GAL4 | the Bloomington *Drosophila* Stock Center | [11] |
| UAS-tau RNAi (HM05101) | the Bloomington *Drosophila* Stock Center | TRiP at Harvard Medical School |
| UAS-luciferase (JF01801) ( used as a control strain for milton RNAi ^TRiP^ in some of the experiments ) | the Bloomington *Drosophila* Stock Center | TRiP at Harvard Medical School |

**Table S1. Fly stocks.**

**References for Table S1**

1. Wittmann CW, Wszolek MF, Shulman JM, Salvaterra PM, Lewis J, et al. (2001) Tauopathy in Drosophila: neurodegeneration without neurofibrillary tangles. Science 293: 711-714.

2. Freeman M (1996) Reiterative use of the EGF receptor triggers differentiation of all cell types in the Drosophila eye. Cell 87: 651-660.

3. Dietzl G, Chen D, Schnorrer F, Su KC, Barinova Y, et al. (2007) A genome-wide transgenic RNAi library for conditional gene inactivation in Drosophila. Nature 448: 151-156.

4. Iijima-Ando K, Hearn SA, Shenton C, Gatt A, Zhao L, et al. (2009) Mitochondrial Mislocalization Underlies Aβ42-Induced Neuronal Dysfunction in a Drosophila Model of Alzheimer’s Disease. PLoS ONE 4: e8310.

5. Liu S, Sawada T, Lee S, Yu W, Silverio G, et al. (2012) Parkinson's Disease-Associated Kinase PINK1 Regulates Miro Protein Level and Axonal Transport of Mitochondria. PLoS Genet 8: e1002537.

6. McDonald JA, Khodyakova A, Aranjuez G, Dudley C, Montell DJ (2008) PAR-1 kinase regulates epithelial detachment and directional protrusion of migrating border cells. Curr Biol 18: 1659-1667.

7. Iijima-Ando K, Zhao L, Gatt A, Shenton C, Iijima K (2010) A DNA damage-activated checkpoint kinase phosphorylates tau and enhances tau-induced neurodegeneration. Hum Mol Genet 19: 1930-1938.

8. Wang JW, Imai Y, Lu B (2007) Activation of PAR-1 kinase and stimulation of tau phosphorylation by diverse signals require the tumor suppressor protein LKB1. J Neurosci 27: 574-581.

9. Kim M, Lee JH, Koh H, Lee SY, Jang C, et al. (2006) Inhibition of ERK-MAP kinase signaling by RSK during Drosophila development. EMBO J 25: 3056-3067.

10. Fossgreen A, Bruckner B, Czech C, Masters CL, Beyreuther K, et al. (1998) Transgenic Drosophila expressing human amyloid precursor protein show gamma-secretase activity and a blistered-wing phenotype. Proc Natl Acad Sci U S A 95: 13703-13708.

11. Lin DM, Goodman CS (1994) Ectopic and increased expression of Fasciclin II alters motoneuron growth cone guidance. Neuron 13: 507-523.
