# Supplementary material for: Loss of Axonal Mitochondria Promotes Tau-Mediated Neurodegeneration and Alzheimer's Disease–Related Tau Phosphorylation Via PAR-1
Source: PLoS Genet. 2012 Aug 30;8(8):e1002918. doi: 10.1371/journal.pgen.1002918 (PMC3431335; doi:10.1371/journal.pgen.1002918)
Supplement: Table S2 — Genotypes of the flies that were used in each experiment. (DOC) [file pgen.1002918.s015.doc]

| Figure 1 | milton RNAi^GD^ | UAS-milton RNAi^GD^/+; gmr-GAL4/+;+/+ |
| --- | --- | --- |
|  | control | +/+;gmr-GAL4/+;+/+ |
|  | tau | +/+;gmr-GAL4/+;UAS-tau/+ |
|  | tau+milton RNAi^GD^ | UAS-milton RNAi^GD^/+;gmr-GAL4/+;UAS-tau/+ |
|  | tau+milton RNAi^TRiP^ | +/+;gmr-GAL4/+;UAS-tau/UAS-milton RNAi^TRiP^ |
|  | tau+Miro RNAi^KK^ | +/+;gmr-GAL4/UAS-Miro RNAi^KK^;UAS-tau/+ |
|  | tau+Miro RNAi^iai^ | +/+;gmr-GAL4/+;UAS-tau/UAS-Miro RNAi^iai^ |
|  | Miro RNAi^iai^ | +/+;gmr-GAL4/+;UAS-Miro RNAi^iai^ /+ |
|  | tau+luciferase RNAi | +/+;gmr-GAL4/UAS-luciferase RNAi;UAS-tau/+, |
| Figure 2 | tau | +/+;gmr-GAL4/+;UAS-tau/+ |
|  | tau+milton RNAi^GD^ | UAS-milton RNAi^GD^/+;gmr-GAL4/+;UAS-tau/+ |
|  | control | +/+;gmr-GAL4/+;+/+ |
|  | milton RNAi^GD^ | UAS-Milton RNAi^GD^/+;gmr-GAL4/+;+/+ |
| Figure 3 | tau | +/+;gmr-GAL4/+;UAS-tau/+ |
|  | tau+milton RNAi^GD^ | UAS-milton RNAi^GD^/+;gmr-GAL4/+;UAS-tau/+ |
|  | tau+ MiroRNAi^KK^ | +/+;gmr-GAL4/UAS-MiroRNAi^KK^;UAS-tau/+ |
| Figure 4 | tau | +/+;gmr-GAL4/+;UAS-tau/+ |
|  | tau+PAR-1 RNAi | +/+;gmr-GAL4/+;UAS-tau/UAS-PAR-1 RNAi |
|  | tau+PAR-1 RNAi+milton RNAi^GD^ | UAS-milton RNAi^GD^ /+;gmr-GAL4/+;UAS-tau/UAS-PAR-1 RNAi |
| Figure 5 | tau+milton RNAi^GD^ | UAS-Milton RNAi^GD^/+;gmr-GAL4/+;UAS-tau/+ |
|  | tau+milton RNAi^GD^+PAR-1 RNAi | UAS-Milton RNAi^GD^/+;gmr-GAL4/+;UAS-tau/UAS-PAR-1 RNAi |
|  | tau+milton RNAi^GD^+luciferase RNAi | UAS-Milton RNAi^GD^/+;gmr-GAL4/UAS-luciferase RNAi;UAS-tau/+ |
|  | tau | +/+;gmr-GAL4/+;UAS-tau/+ |
|  | S262Atau | +/+;gmr-GAL4/+;UAS-S262Atau/+ |
|  | tau+milton RNAi^GD^ | UAS-milton RNAi^GD^/+;gmr-GAL4/+;UAS-tau/+ |
|  | S262Atau+milton RNAi^GD^ | UAS-milton RNAi^GD^/+;gmr-GAL4/+;UAS-S262Atau/+ |
| Figure 6 | PAR-1 in A | +/+;gmr-GAL4/+;UAS-PAR-1-myc/+ |
|  | PAR-1+milton RNAi^GD^ | UAS-Milton RNAi^GD^/+;gmr-GAL4/+;UAS-PAR-1-myc/+ |
|  | PAR-1 in B | +/+;gmr-GAL4/+;UAS-PAR-1myc/UAS-luciferase |
|  | PAR-1+milton RNAi^TRiP^ | +/+;gmr-GAL4/+;UAS-PAR-1myc/UAS-milton RNAi^TRiP^ |
|  | PAR-1 in C | +/+;gmr-GAL4/+;UAS-PAR-1-myc/+ |
|  | PAR-1+Miro RNAi^KK^ | +/+;gmr-GAL4/UAS-Miro RNAi^KK^;UAS-PAR-1myc/+ |
|  | PAR-1 in D | +/+;gmr-GAL4/+;UAS-PAR-1-myc/+ |
|  | PAR-1+Luciferase RNAi | +/+;gmr-GAL4/UAS-luciferase RNAi;UAS-PAR-1myc/+ |
|  | PAR-1 T408A | +/+;gmr-GAL4/+;UAS-PAR-1 T408A-myc/+ |
|  | PAR-1 T408A+milton RNAi^GD^ | UAS-Milton RNAi^GD^ /+;gmr-GAL4/+;UAS-PAR-1 T408A-myc/+ |
| Figure 7 | milton RNAi^GD^ in A | UAS-Milton RNAi^GD^/elav-GAL4; gmr-GAL4/+;+/+ |
|  | control in B | elav-GAL4/+;gmr-GAL4/UAS-luciferase RNAi;+/+ |
|  | milton RNAi^GD^ in C, D and E | UAS-Milton RNAi^GD^/+; gmr-GAL4/+;+/+ |
|  | control in F | +/+;gmr-GAL4/+;+/+ |
|  | milton RNAi^TRiP^ in H and I | elav-GAL4/+; gmr-GAL4/+;UAS-milton RNAi^TRiP^/+ |
|  | control in J | elav-GAL4/+;gmr-GAL4/+;UAS-luciferase/+ |
|  | Miro RNAi^KK^ in L | +/+;gmr-GAL4/UAS-MiroRNAi^KK^;+/+ |
|  | control in M | +/+;gmr-GAL4/+;+/+ |
| Figure 8 | milton RNAi^GD^ in A and E | UAS-milton RNAi^GD^/+; gmr-GAL4/+;+/+ |
|  | milton RNAi^GD^+tau RNAi | UAS-milton RNAi^GD^/+;gmr-GAL4/+;UAS-tauRNAi/+ |
|  | milton RNAi^GD^+luciferase RNAi | UAS-milton RNAi^GD^/+;gmr-GAL4/UAS-luciferase RNAi;+/+ |
|  | milton RNAi^GD^+PAR-1 RNAi | UAS-milton RNAi^GD^/+;gmr-GAL4/+;UAS-PAR-1 RNAi/+ |

**Table S2. Fly genotypes.**
