# Supplementary material for: Loss of Axonal Mitochondria Promotes Tau-Mediated Neurodegeneration and Alzheimer's Disease–Related Tau Phosphorylation Via PAR-1
Source: PLoS Genet. 2012 Aug 30;8(8):e1002918. doi: 10.1371/journal.pgen.1002918 (PMC3431335; doi:10.1371/journal.pgen.1002918)
Supplement: Text S1 — Materials and methods for mito-GFP analysis in fly brains. (DOC) [file pgen.1002918.s016.doc]

**Text S1. Supporting Materials and Methods**

**Mito-GFP analysis in fly brains.** Fly brains were dissected in cold PBS, fixed in PBS containing 4% paraformaldehyde (Electron Microscopy Sciences), and then placed under vacuum in PBS containing 4% paraformaldehyde and 0.25% Triton X-100. The fluorescence intensity in the mushroom body regions was analyzed using a confocal microscope (Carl Zeiss LSM 510) and quantified using NIH image.
